# Early life interventions metformin and trodusquemine metabolically reprogram the developing mouse liver through transcriptomic alterations

**DOI:** 10.1111/acel.14227

**Published:** 2024-05-27

**Authors:** Sarah A. Ashiqueali, Augusto Schneider, Xiang Zhu, Ewelina Juszczyk, Mishfak A. M. Mansoor, Yun Zhu, Yimin Fang, Bianka M. Zanini, Driele N. Garcia, Natalie Hayslip, David Medina, Samuel McFadden, Robert Stockwell, Rong Yuan, Andrzej Bartke, Michael Zasloff, Shadab Siddiqi, Michal M. Masternak

**Affiliations:** ^1^ Burnett School of Biomedical Sciences University of Central Florida College of Medicine Orlando Florida USA; ^2^ Faculdade de Nutrição Universidade Federal de Pelotas Pelotas Brazil; ^3^ Research & Development Center Celon Pharma S.A. Kazun Nowy Poland; ^4^ Department of Internal Medicine Southern Illinois University School of Medicine Springfield Illinois USA; ^5^ MedStar Georgetown Transplant Institute Georgetown University School of Medicine Washington DC USA; ^6^ Department of Head and Neck Surgery Poznan University of Medical Sciences Poznan Poland

**Keywords:** development, early life interventions, juvenile mice, lifespan and healthspan, liver metabolism, metformin, microRNAs and mRNAs, postnatal window, trodusquemine (3‐N‐1(spermine)‐7, 24‐dihydroxy‐5‐cholestane 24‐sulfate)

## Abstract

Recent studies have demonstrated the remarkable potential of early life intervention strategies at influencing the course of postnatal development, thereby offering exciting possibilities for enhancing longevity and improving overall health. Metformin (MF), an FDA‐approved medication for type II diabetes mellitus, has recently gained attention for its promising anti‐aging properties, acting as a calorie restriction mimetic, and delaying precocious puberty. Additionally, trodusquemine (MSI‐1436), an investigational drug, has been shown to combat obesity and metabolic disorders by inhibiting the enzyme protein tyrosine phosphatase 1b (*Ptp1b*), consequently reducing hepatic lipogenesis and counteracting insulin and leptin resistance. In this study, we aimed to further explore the effects of these compounds on young, developing mice to uncover biomolecular signatures that are central to liver metabolic processes. We found that MSI‐1436 more potently alters mRNA and miRNA expression in the liver compared with MF, with bioinformatic analysis suggesting that cohorts of differentially expressed miRNAs inhibit the action of phosphoinositide 3‐kinase (*Pi3k*), protein kinase B (*Akt*), and mammalian target of rapamycin (*Mtor*) to regulate the downstream processes of de novo lipogenesis, fatty acid oxidation, very‐low‐density lipoprotein transport, and cholesterol biosynthesis and efflux. In summary, our study demonstrates that administering these compounds during the postnatal window metabolically reprograms the liver through induction of potent epigenetic changes in the transcriptome, potentially forestalling the onset of age‐related diseases and enhancing longevity. Future studies are necessary to determine the impacts on lifespan and overall quality of life.

## Graphical abstract

This study examined the effects of metformin (MF) and trodusquemine (MSI‐1436) on the livers of young, developing mice. We discovered that MSI‐1436 significantly alters mRNA and miRNA expression more potently than MF, impacting key metabolic pathways. These findings suggest that these early life interventions can metabolically reprogram the liver during the postnatal window through significant epigenetic modifications, thereby potentially forestalling the onset of age‐related diseases to promote healthspan and extend lifespan.

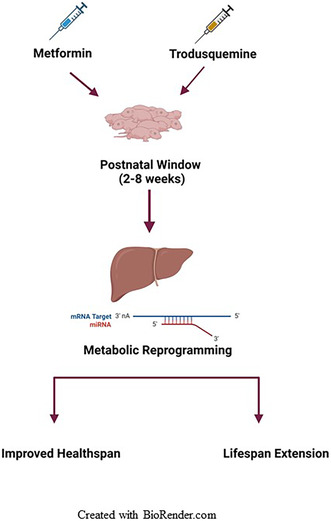

**Abbreviations**
*Abcg5*
: ATP binding‐cassette sub family g member 5
*Acc*
: Acetyl‐CoA‐carboxylase 1
*Akt*
: Protein kinase B
*Ampk*
: Adenosine monophosphate‐activated protein kinase
ANOVA: Analysis of variance
*Apoa1*
: Apolipoprotein a1
*Apoa4*
: Apolipoprotein a4
*Apob*
: Apolipoprotein b
*Apoe*
: Apolipoprotein e
AUC: Area under the curve
*Cd36*
: Cluster of differentiation 36
cDNA: Complementary DNA
*Cideb*
: Cell death‐inducing DFFA like effector B
*Cpt1*
: Carnitine palmitoyltransferase 1
CR: Calorie restriction
Ct: Cycle of threshold
CTL: Control
*Cyp7a1*
: Cholesterol 7‐alpha hydroxylase
Dim: Dimension
FC: Fold change
FDA: Food and Drug Administration
FDR: False discovery rate
*Foxo*
: Forkhead box protein
GH: Growth hormone
GnRH: Gonadotropin‐releasing hormone
GTT: Glucose tolerance test
*HmgCoA*
: 3‐hydroxy‐3‐methylglutaryl coenzyme A
i.p.: Intraperitoneal
*Igf1*
: Insulin‐like growth factor 1
ITT: Insulin tolerance test
KEGG: Kyoto encyclopedia of genes and genomes
*Lxr*
: Liver x receptor
MF: Metformin
miRNA: Microribonucleic acid
mRNA: Messenger ribonucleic acid
MSI‐1436: Trodusquemine
*Mtor*
: Mammalian target of rapamycin
*Mttp*
: Microsomal triglyceride transfer protein
NAFLD: Non‐alcoholic fatty liver disease
PCA: Principal component analysis
*Pi3k*
: Phosphoinositide 3‐kinase
PPAR: Peroxisome proliferator‐activated receptor
*Ptp1b*
: Proteine tyrosine phosphatase 1b
qPCR: Quantitative polymerase chain reaction
RNA: Ribonucelic acid
*Sar1a*
: Secretion associated Ras related GTPase 1a
*Sar1b*
: Secretion associated Ras related GTPase 1b
*Scd1*
: Stearoyl‐Coenzyme A desaturase‐1
*Sec22b*
: Sec22 homolog b vesicle trafficking protein
SEM: Standard error of mean
*Sirt1*
: Sirtuin 1
*Srebp1*
: Sterol regulatory element‐binding protein 1
*Stx5a*
: Syntaxin 5a
T2DM: Type 2 diabetes mellitus
TODAY: Treatment options for type 2 diabetes in adolescents and youth
UM‐HET3: University of Michigan genetically heterogeneous
VLDL: Very‐low density lipoprotein
*β2M*
: Beta‐2 microglobulin

## 1 INTRODUCTION

The “silver tsunami” looms on the horizon, with the number of people aged 60 and older projected to double by the year 2050 (Mitchell, 2014; World Health Organization [WHO], 2022). While the world continues to witness a remarkable increase in the aging population due to considerable advances in medicine, there is an urgent demand for novel strategies to improve the quality of life in these extended years. A key determinant of extended lifespan is delayed puberty which is believed to diminish the risk of mortality by correspondingly preventing the onset of many age‐related manifestations including neurodegeneration, type II diabetes, cardiovascular disease, nonalcoholic fatty liver disease (NAFLD), and cancer (Aguiar‐Oliveira & Bartke, 2019; Shadyab et al., 2017; Widen et al., 2012). As such, the period of postnatal development is regarded as a critical stage during an organism's lifetime due to its influence on the future trajectory for health and vitality (Dorn et al., 2019; McMullen & Mostyn, 2009; Zhu et al., 2022). Previous studies have elucidated that early life intervention methods such as growth hormone replacement therapy, food restriction, impaired ghrelin action, manipulation of litter size, and odor priming have significant effects on growth rate, age of sexual maturity, and mortality in young mice (Avila et al., 2024; Bartke et al., 2022; Fulton et al., 2024; Garratt et al., 2022; Parra‐Vargas et al., 2023; Steculorum et al., 2015; Sun et al., 2009; Tong & D'Alessio, 2015). Given that accumulating evidence points out that postnatal conditions in juvenile mice can dictate the adult phenotype, discovering and exploring new early life therapies is a promising avenue for not only increasing the lifespan but also forestalling age‐related diseases.

The perinatal transition is characterized by crucial physiological adaptations that determine the overall well‐being of the newborn (Gruppuso & Sanders, 2016). These changes are particularly modulated by the actions of growth hormone (GH) on somatomedins, such as insulin‐like growth factor 1 (*Igf‐1*), which are synthesized in the liver, the major metabolic organ responsible for orchestrating growth and development (Blum et al., 2018; Hyatt et al., 2008). Liver‐derived *Igf‐1* mediates sexual dimorphism, lipid metabolism, and glucose homeostasis, exerting its effects not only locally but also systemically by entering the circulation to promote cell proliferation and survival in peripheral tissues (Vazquez‐Borrego et al., 2021). The activation of downstream signal transduction mechanisms such as the phosphatidylinositol‐3‐kinase/protein kinase B (*Pi3k/Akt/Mtor*) pathway by *Igf‐1* underscores its pivotal role in the modulation of several important anabolic and catabolic processes (Liu et al., 2016). Studies of long‐living organisms such as Snell and Ames dwarf mouse mutants have demonstrated that deficiencies in GH and circulating *Igf1* contribute to their small size and improved healthspan and lifespan (Ashpole et al., 2017; Brown‐Borg & Bartke, 2012; Sharp & Bartke, 2005; Sun et al., 2017). Conversely, early life GH‐replacement therapy for 6 weeks, amounting for less than 5% of total lifespan, negatively modulated longevity in long‐living Ames dwarf mice (Panici et al., 2010).

Early life interventions have several remarkable long‐term implications on healthspan and lifespan, influencing metabolic reprogramming, immune development, cognitive function, and biological aging (Bartke et al., 2022; Gollwitzer & Marsland, 2015; Mika et al., 2017). Namely, the postnatal window is characterized by critical physiological adjustments important for healthy development as the newborn transitions from the intrauterine to extrauterine environment (Cameron & Demerath, 2002). Developmental plasticity during this time results in major changes across several organ systems altering health outcomes and laying the foundation for future vitality. Particularly, epigenetic changes during early life can influence gene expression and therefore have long‐lasting effects on health and disease (Wang et al., 2013). These long‐lasting changes can reduce the economic and social burdens of chronic disease, further underscoring the importance of the postnatal window due to its impact on lifelong health. Given the growing prevalence of childhood obesity, it is imperative to understand how these key biological pathways may be dysregulated (Marcus et al., 2022). As such, elucidating the impacts of pharmacological agents on the transcriptome during the postnatal period is vital for pioneering novel early life interventions which can extend lifespan and improve healthspan through targeted modulation of the GH/*Igf* axis and the *Pi3k/Akt/Mtor* signaling pathway (Aguiar‐Oliveira & Bartke, 2019; Brown‐Borg & Bartke, 2012).

Recently, metformin (MF), an FDA‐approved biguanide that is typically used to treat type II diabetes mellitus (T2DM), has garnered notable attention for its potential anti‐aging properties which are being actively investigated in ongoing clinical trials (Al‐Kuraishy et al., 2015, 2020, 2023; Glossmann & Lutz, 2019; Martin‐Montalvo et al., 2013; Novelle et al., 2016). Particularly, it has been demonstrated that MF has the remarkable ability to act as a calorie restriction (CR) mimetic (Ingram et al., 2006; Martin‐Montalvo et al., 2013). This is achieved through activation of the cellular energy sensor adenosine monophosphate‐activated protein kinase (*Ampk*), a key regulator of lipid and glucose catabolism (Madiraju et al., 2014). Calorie restriction has been demonstrated to suppress the *Igf/Pi3k/Akt/Mtor* pathway thereby resulting in metabolic alterations and transcriptomic changes that support healthy aging and promote lifespan extension (Karagöz & Gülçin Sağdıçoğlu Celep, 2023; Mercken et al., 2013). It has also been shown that MF can delay precocious puberty in girls, which may have profoundly positive impacts on both lifespan and healthspan (Ibanez, et al., 2011a, 2011b; Zhu et al., 2022).

Concurrently, trodusquemine (MSI‐1436), an aminosterol initially isolated from the dogfish shark, has emerged as a novel candidate to treat obesity and other metabolic disorders (Ahima et al., 2002; Bourebaba, Serwotka‐Suszczak, Bourebaba, et al., 2023; Bourebaba, Serwotka‐Suszczak, Pielok, et al., 2023; Lantz et al., 2010; Pandey et al., 2014; Smith et al., 2017; Zasloff, William, et al., 2001). Namely, MSI‐1436 is a naturally occurring inhibitor of central and peripheral protein tyrosine phosphatase 1B (*Ptp1b*) which has been shown to modulate insulin, leptin, GH, and *Igf1* action (Bourebaba, Serwotka‐Suszczak, Bourebaba, et al., 2023; Bourebaba, Serwotka‐Suszczak, Pielok, et al., 2023; Lantz et al., 2010; Owen et al., 2015). This enzyme is known to dephosphorylate the insulin receptor which can lead to subsequent insulin and leptin resistance (Bence et al., 2006; Delibegovic et al., 2009; Feldhammer et al., 2013; Zabolotny et al., 2002). While several studies demonstrate that MSI‐1436 reduces hepatic lipogenesis and counteracts oxidative stress, its effects in young animals and its broader implications on aging are yet to be investigated (Bourebaba, Serwotka‐Suszczak, Pielok, et al., 2023; Takahashi et al., 2004).

Our study focuses on the impact of both MF and MSI‐1436 on the livers of heterogenous juvenile UM‐HET3 male and female mice, a diverse strain commonly used by the National Institute of Aging to evaluate life extension treatments (Zhu et al., 2022). We aimed to understand how these compounds, when administered in early life, can influence mRNA signatures integral to the processes of postnatal liver development and metabolism. Alongside, we conducted extensive profiling of small, noncoding miRNAs given their robust ability to control gene expression, thereby modulating signaling pathways that can influence various disease states (Cai et al., 2009). In summary, our study shows that treatment of young mice with these compounds has profound effects on the metabolic phenotype of the liver, with further research required to determine how such early interventions impact both longevity and quality of life.

## 2 METHODS

### 2.1 Study approval

Animal protocol was approved by the Southern Illinois University Institutional Animal Care and Use Committee. Using 10 breeding pairs from The Jackson Laboratory (Bar Harbor, Maine 04609), female CB6F1/J (#100007) and male C2D2F1/J (#100004) mice were bred to create UM‐HET3 mice, a genetically heterogenous cohort that better represents human populations (Zhu et al., 2022). Of note, these mice are used in the National Institutes on Aging's Interventions Testing Program (National Institute on Aging, n.d.; Zhu et al., 2022).

### 2.2 Experimental design

Mice were housed under a 12:12 h light: dark cycle at 22 ± 1°C with 35%–50% relative humidity and fed ad libitum (Cat. # 5001, LabDiet PMI Feeds; 23.5% protein, 5% fat, and 5.8% crude fiber). At 2 weeks of age, both male and female mice (*n* = 10 per group) were randomly separated into the following experimental groups: (1) vehicle saline‐treated controls, (2) 100 mg/kg metformin (MF) treated, and (3) 2 mg/kg trodusquemine (MSI‐1436) treated. Doses of metformin were determined based on FDA guidelines and previously conducted in vivo studies (Ahima et al., 2002; LaMoia & Shulman, 2021). The dose of MSI‐1436 chosen was known to be well tolerated by neonatal mice (M. Zasloff, unpublished observations). The duration of treatment, from Days 15 to 56, was decided based on previous studies where growth hormone intervention and metformin treatment have shown significant alterations in development and metabolic traits (Martinez et al., 2015; Sun et al., 2017; Zhu et al., 2022). Importantly, previous studies suggest that interventions during early life, which alter development and metabolic traits, may have long‐lasting effects on aging and lifespan (Aparicio‐Puerta et al., 2022; Ge et al., 2020; Sun et al., 2009, 2017). Gavage feeding is dangerous during the neonatal period due to the sensitivity of the upper gastrointestinal tract. Alternatively, supplementation of food with metformin would confound the study due to disparities in milk consumption thus yielding variable dosing between animals (Zhu et al., 2022). Mice were administered either daily intraperitoneal (i.p.) injections of MF or triweekly i.p., injections of MSI‐1436 for a period of 6 consecutive weeks. At 8 weeks (2 months) of age, mice were sacrificed by cervical dislocation under isoflurane and liver tissues were collected, weighed, and stored at −80°C.

### 2.3 Glucose tolerance test and insulin tolerance test

Glucose tolerance test (GTT) was conducted 16 h following fasting. Insulin tolerance test (ITT) was performed under non‐fasted conditions. Glucose (1 g/kg) or insulin (1 IU/kg, Sigma, St. Louis, MO, USA) was administered via intraperitoneal (i.p.) injection. Blood glucose levels were measured over 120 min by tail vein using AgaMatrix Wavesense Presto Blood Glucose Monitoring System (AgaMatrix, New Hampshire, 03079) (Zhu et al., 2022). Area under the curve (AUC) was assessed to quantify blood glucose changes.

### 2.4 RNA extraction and cDNA synthesis

Following the manufacturer's protocol, tissue lysis was performed using QIAzol reagent and samples were centrifuged at 12,000 *g* for 15 min at 4°C for isolation of the aqueous phase. Thereafter, RNA was purified and eluted using a spin column. The final products were suspended in 30 μL of RNase‐free water and evaluated for quality and quantity using a spectrophotometer. RNA integrity was assessed by measuring the ratio of absorbance (260 nm/280 nm) with purity values of 1.9 or greater accepted for downstream molecular analysis. Complementary DNA (cDNA) for mRNA was synthesized from 1 μg of total RNA per sample using the iScriptTM cDNA Synthesis Kit. cDNA was diluted to 200 ng for qPCR and subsequently stored at −20°C.

### 2.5 Quantitative polymerase chain reaction (qPCR)

Quantitative PCR (qPCR) was conducted on the Quant Studio 7 System for 40 cycles (annealing/extension at 60°C). qPCR reactions were performed in duplicate using 5 μL SYBR Green Master Mix, 0.2 μL of 10 μM forward primer, 0.2 μL of 10 μM and reverse primer, 12.6 μL nuclease‐free water, and 2 μL of 200 ng cDNA template per sample. Primers for target genes are listed in Table S1. To determine the relative expression of each gene of interest, the delta–delta CT method was employed, with transcript levels normalized to the housekeeping gene Beta‐2 microglobulin (β2M).

### 2.6 miRNA library construction

In total, 1.5 μg of RNA isolated from each liver sample (*n* = 6 per group) was used to generate miRNA sequencing libraries with the NETFLEX® Small RNA‐Seq Kit V3 for Illumina Platforms (PerkinElmer). Sample concentrations and qualities were assessed on the Agilent Bioanalyzer High Sensitivity RNA chip. Thereafter, 3′ and 5′ adapters were gradually ligated to small RNA molecules and ligation products were then reverse transcribed to synthesize single stranded cDNA. First strand synthesis products were amplified with 18 cycles of PCR using NEXTFLEX® universal primer and a unique barcoded primer to allow for sample multiplexing. Following gel‐free cleanup, libraries were assessed using the Agilent Bioanalyzer High Sensitivity DNA chip and samples were then pooled for sequencing on an Illumina HiSeq 2000 instrument (Martinez et al., 2015).

### 2.7 Statistical analysis and bioinformatics

2^−ΔΔCt^ method was used to calculate and normalize gene expression of each target gene, in which −ΔΔCt was calculated according to the formula: −ΔΔCt = average(ΔCt _control sample_) − ΔCt _treated sample_, where ∆Ct = Ct _target gene_ − Ct _housekeeping gene_. β2M was selected as a housekeeping gene, and untreated male animals were assigned as control. To evaluate the effects of treatment and sex on the gene expression, we first performed multi‐factor ANOVA with interaction between treatment and sex included and then conducted Tukey's multiple pairwise comparisons over sex and treatment. A heatmap was produced to visualize the overall gene expression profile by treatment and sex. To further evaluate the contribution of each gene to the overall difference among the different treatments and sexes, we performed principal component analysis (PCA) and visualized the results on PCA biplots. All statistical analyses were performed using Stata MP 15 (StataCorp LLC, 2019), R packages, and GraphPad Prism. All tests were two‐tailed with a significant level of α (type I error) <0.05. Gene enrichment analysis was conducted using ShinyGO 0.77 webtool with FDR cutoff of <0.05 to demonstrate the most highly regulated KEGG pathways associated with the mRNAs investigated (Ge et al., 2020).

Sequencing files were processed using the online tool sRNAtoolbox (Aparicio‐Puerta et al., 2022). Differential expression of miRNAs was conducted in R software by EdgeR package (Robinson et al., 2010). miRNAs with a *p*‐value and FDR < 0.05 and FC > 2.0 were considered upregulated while miRNAs with a *p*‐value and FDR < 0.05 and FC < 0.50 were considered downregulated. Heatmap for all detected miRNAs was generated using ClustVis online webtool (Metsalu & Vilo, 2015). Rows and columns were clustered by correlation distance and average linkage. miRNA functional analysis for validated KEGG and Reactome pathways was conducted using TaRBase v.8.0 gene union and pathway union pipeline on the web‐based platform DIANA tools miRPath v.3.0 and v.4.0 with significant functionally enriched pathways defined by *p*‐value and FDR < 0.05 (Tastsoglou et al., 2023; Vlachos et al., 2015). Alongside, miRPathDB v.2.0 was also used to determine mRNA targets of significantly differentiated miRNAs (Kehl et al., 2020). Summary figure to show the proposed mechanism of action of upregulated miRNAs in response to MSI‐1436 was generated using BioRender.com.

## 3 RESULTS

### 3.1 Effects of MF and MSI‐1436 on physiological parameters in juvenile mice

**FIGURE 1:**
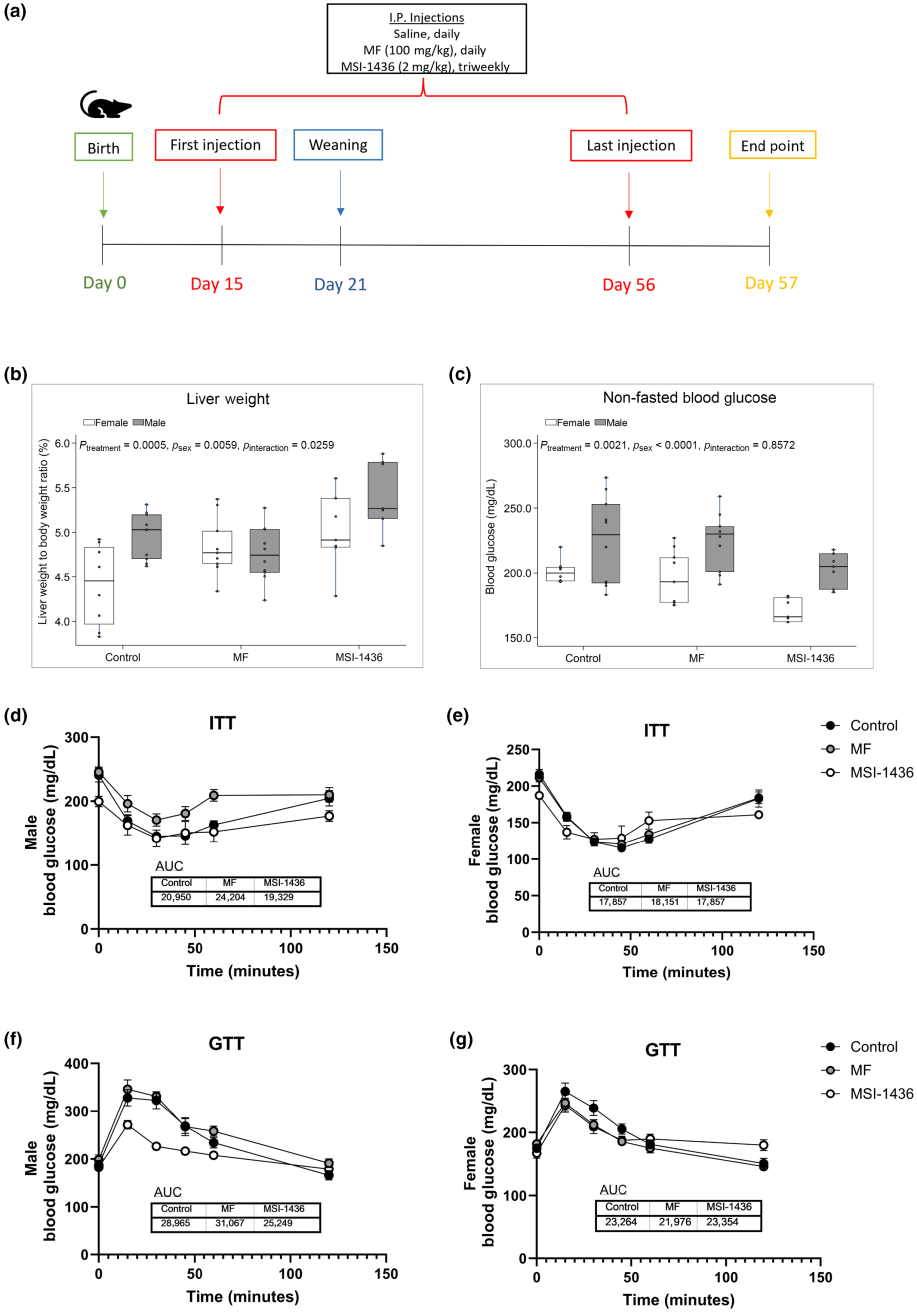
Study timeline and outcomes of physiological parameters assessed. (a) Timeline of the study. UM‐HET3 male and female heterogenous mice were administered saline, 100 mg/kg metformin (MF), or triweekly 2 mg/kg trodusquemine (MSI‐1436) via intraperitoneal (i.p.) injections starting at 15 days of age. Study was conducted over the course of 6 weeks, with mice weaned at 21 days and euthanized at 57 days. (b) Liver weight (g) was normalized to body weight (g) at the end point of the study. Values represent mean ± SEM. (c) Non‐fasted glucose (mg/dL) was assessed at the end point of the study. Values represent mean ± SEM. (d, e) Line graph of glucose and (f, g) insulin tolerance test results in male and female animals, conducted over the course of 120 min, with AUC assessed to quantify glucose changes. Values represent mean ± SEM.

Mice of both sexes were treated with either vehicle, MF, or MSI‐1436 for 6 weeks beginning at 2 weeks of age (Figure 1a). Liver weight to body weight ratio was assessed with MSI‐1436 increasing gross liver weight in both male and female animals, and overall effect of treatment (*p* = 0.0005), sex (*p* = 0.0059), and interaction between sex and treatment (*p* = 0.0259) observed (Figure 1b). Non‐fasted glucose decreased after MSI‐1436 treatment, with overall effect of treatment (*p* = 0.0021) and sex (*p* < 0.0001) observed (Figure 1c). Alongside, insulin (ITT) and glucose tolerance tests (GTT) were conducted, with AUC assessed to quantify blood glucose changes. ITT results demonstrate lower non‐fasted basal glucose levels (at 0 min) in both male and female MSI‐1436‐treated animals against the CTL‐ and MF‐treated animals (Figure 1d,e). MSI‐1436 did not alter ITT compared with CTL animals (Figure 1d,e). MF‐treated male animals demonstrated impaired insulin tolerance compared with the CTL‐ and MSI‐1436‐treated male animals (Figure 1d), with minimal changes observed in MF‐treated female animals (Figure 1e). GTT results indicate improved glucose tolerance in male mice following MSI‐1436 treatment and minimal changes in female mice compared with CTL male and female mice, respectively (Figure 1f,g). MF appeared to impair glucose tolerance in the male mice (Figure 1f) while improving glucose tolerance in the female mice (Figure 1g) compared with both control (CTL) and MSI‐1436‐treated mice.

### 3.2 Biomolecular effects of MF and MSI‐1436 on the livers of juvenile mice

**FIGURE 2:**
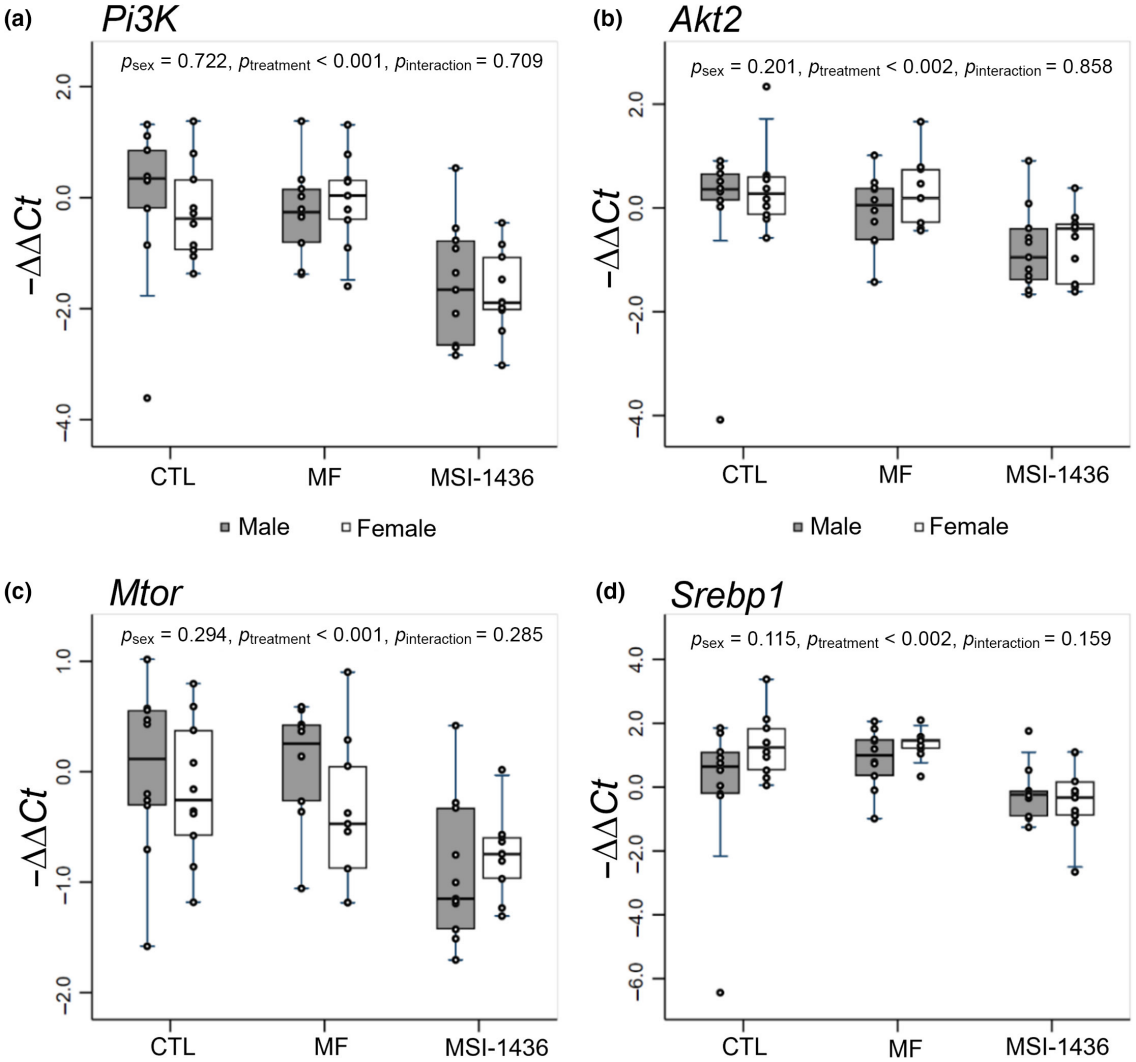
qPCR analysis of mRNAs involved with growth and metabolism. −ΔΔCt method was used to calculate and normalize gene expression of each target gene, with β2M selected as a housekeeping gene and untreated male animals assigned as control samples. Stata MP15 and R packages were used to conduct statistical analysis and generate box‐and‐whisker plots. Multi‐factor ANOVA was performed with interaction between treatment and sex included, and post hoc analysis was conducted using Tukey's multiple pairwise comparisons over sex and treatment.

**FIGURE 3:**
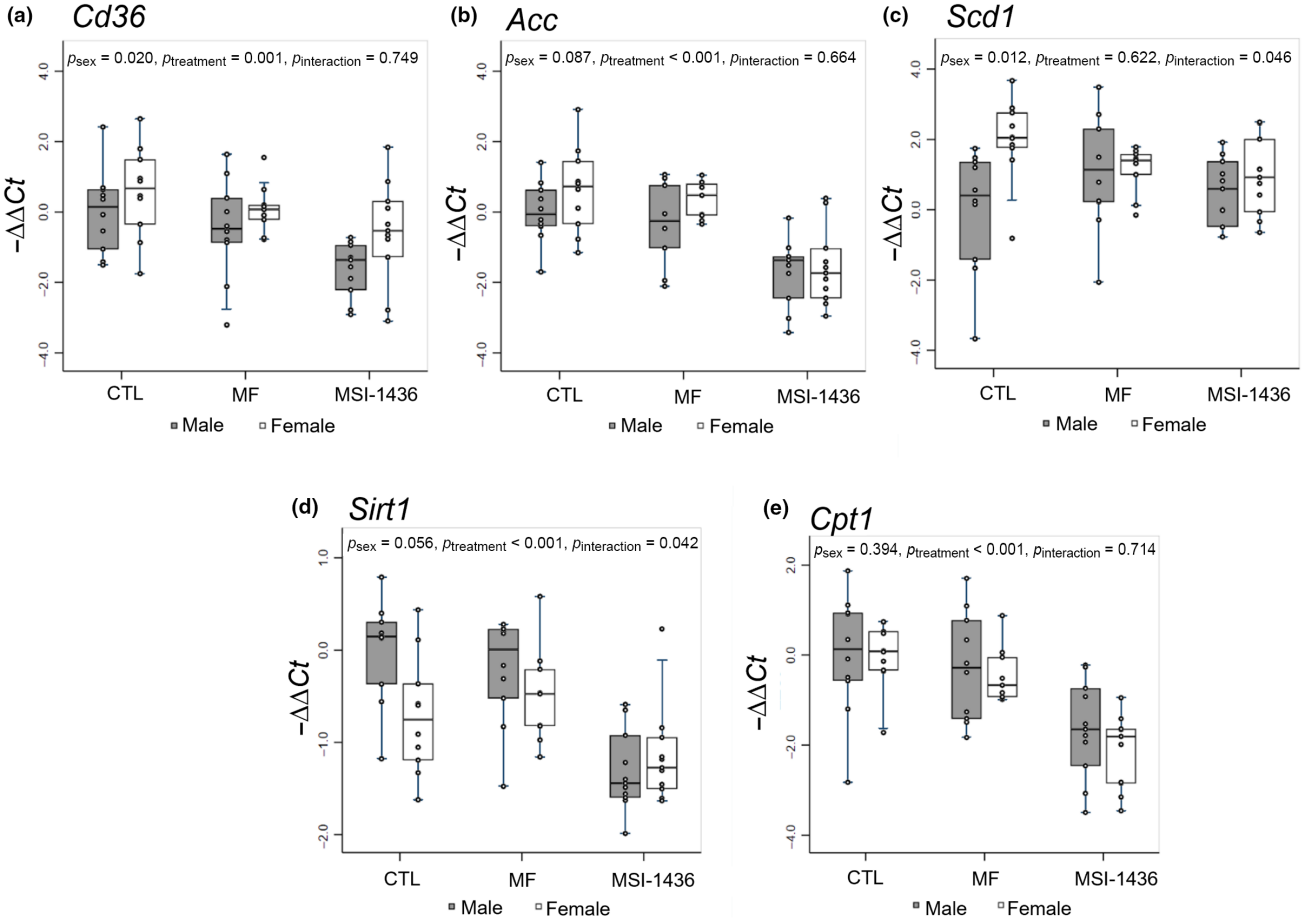
qPCR analysis of mRNAs involved with fatty acid uptake, lipogenesis, and fatty acid oxidation. −ΔΔCt method was used to calculate and normalize gene expression of each target gene, with β2M selected as a housekeeping gene and untreated male animals assigned as control samples. Stata MP15 and R packages were used to conduct statistical analysis and generate box‐and‐whisker plots. Multi‐factor ANOVA was performed with interaction between treatment and sex included, and post hoc analysis was conducted using Tukey's multiple pairwise comparisons over sex and treatment.

**FIGURE 4:**
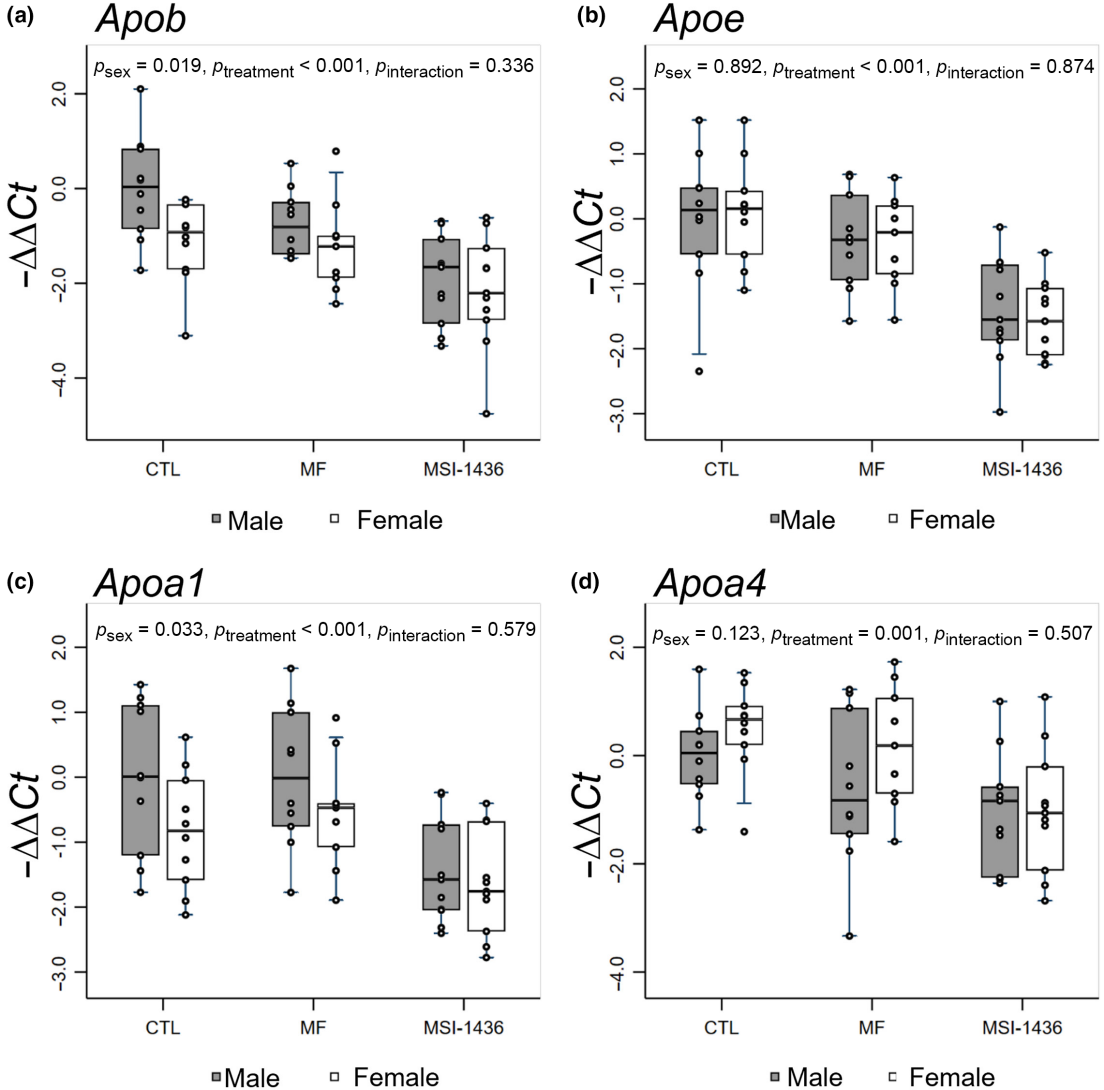
qPCR analysis of mRNAs involved with cholesterol transport. −ΔΔCt method was used to calculate and normalize gene expression of each target gene, with β2M selected as a housekeeping gene and untreated male animals assigned as control samples. Stata MP15 and R packages were used to conduct statistical analysis and generate box‐and‐whisker plots. Multi‐factor ANOVA was performed with interaction between treatment and sex included, and post hoc analysis was conducted using Tukey's multiple pairwise comparisons over sex and treatment.

**FIGURE 5:**
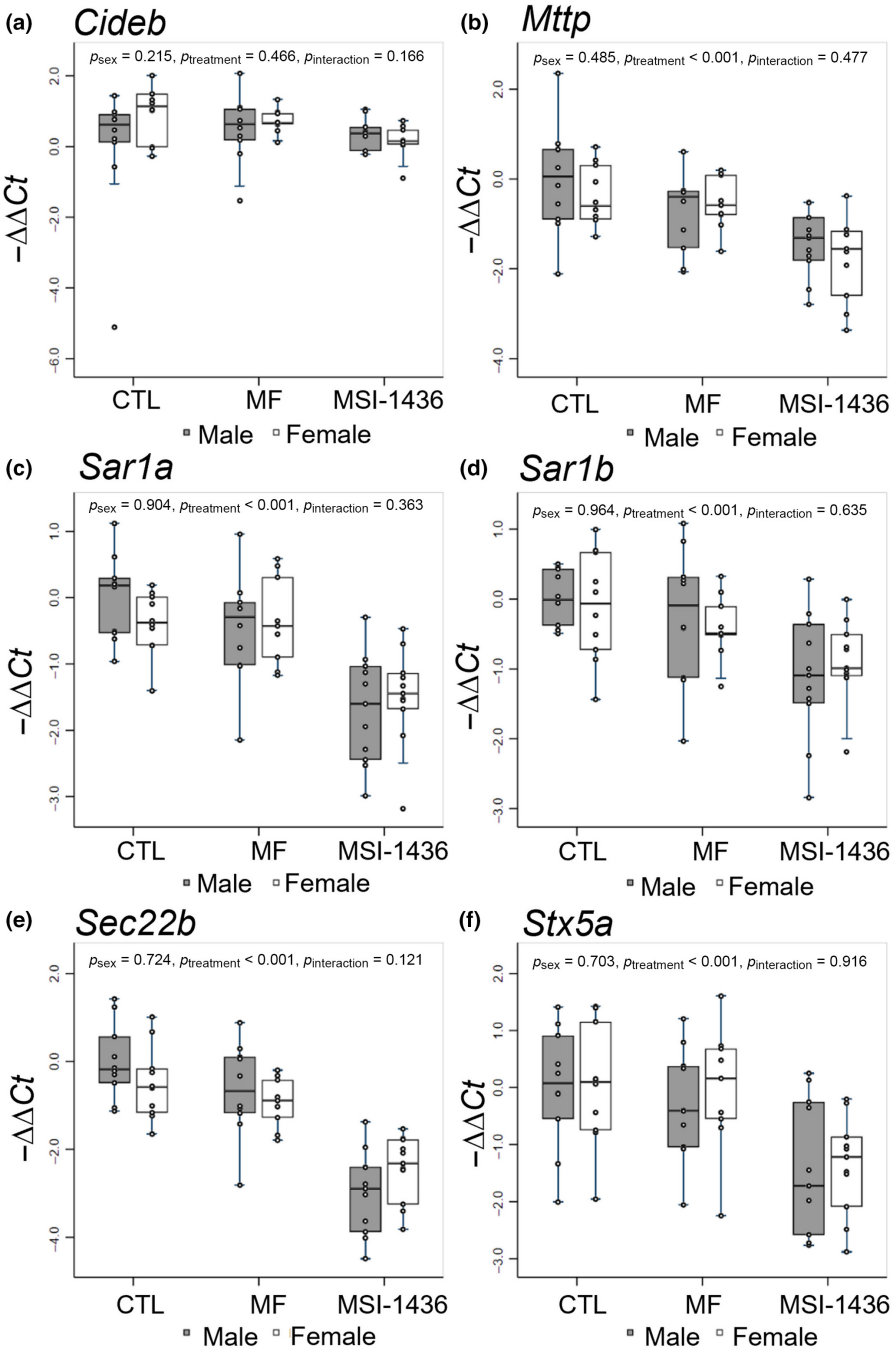
qPCR analysis of mRNAs involved with very‐low‐density lipoprotein transport. −ΔΔCt method was used to calculate and normalize gene expression of each target gene, with β2M selected as a housekeeping gene and untreated male animals assigned as control samples. Stata MP15 and R packages were used to conduct statistical analysis and generate box‐and‐whisker plots. Multi‐factor ANOVA was performed with interaction between treatment and sex included, and post hoc analysis was conducted using Tukey's multiple pairwise comparisons over sex and treatment.

To understand the impacts of MF and MSI‐1436 on metabolic phenotypes, we performed qPCR to measure changes in the relative expression of mRNAs associated with cell growth and metabolism (*Pi3k*, *Akt2*, *Mtor*, *Srebp1*), fatty acid uptake and de novo lipogenesis (*Cd36*, *Acc*, *Scd1*), energy sensing and fatty acid oxidation (*Sirt1*, *Cpt1*), cholesterol transport (*Apob*, *Apoe*, *Apoa1*, *Apoa4*), very‐low‐density lipoprotein transport (*Cideb*, *Mttp*, *Sar1a*, *Sar1b*, *Sec22b*, *Stx5a*), and cholesterol metabolism (*Lxr*, *Abcg5*, *Cyp7a1*). Using multi‐factor ANOVA with post hoc Tukey multiple comparisons test, we examined the modulatory effects of MF and MSI‐1436 on these key mRNAs which are associated with hepatic homeostasis, investigating how both sex and treatment can regulate these molecular pathways (Figures 2, 3, 4, 5; Figure S1 and Table S2).

### 3.3 Expression of mRNAs associated with cell growth and metabolism in the livers of MF and MSI‐1436 treated juvenile mice

In our investigation, we scrutinized the expression of mRNAs (*Pi3k*, *Akt2*, *Mtor*, *Srebp1*) which are integral to growth, survival, and metabolic processes (Figure 2). We noted a discernible downregulation of *Pi3k* in both the male and female MSI‐1436‐treated animals, underscored by a significant effect imparted by treatment (*p* < 0.001) (Figure 2a). In contrast, no notable differences in *Pi3k* were observed in the MF‐treated mice. Similarly, *Akt2* was also significantly downregulated in both the MSI‐1436‐treated male and female animals, with a significant effect of treatment noted (*p* < 0.002) (Figure 2b). Once again, no notable alteration in *Akt2* was observed in the MF‐treated mice. Furthermore, *Mtor* was downregulated in both the MSI‐1436‐treated male and female groups relative to the CTL‐ and MF‐treated male mice, respectively, with overall significant effect of treatment noted (*p* < 0.001) (Figure 2c). Lastly, *Srebp1* was downregulated in MSI‐1436‐treated male and female mice against both CTL‐ and MF‐treated male and female animals with a significant effect of treatment observed (*p* < 0.002) (Figure 2d). Subsequent analysis using Tukey's pairwise comparisons showed that *Srebp1* downregulation was especially pronounced in female MSI‐1436 treated in comparison with female control and MF‐treated animals (*p* = 0.032, *p* = 0.036, respectively) (Figure 2d; Table S2). No significant changes were observed in both the male and female MF‐treated animals (Figure 2d).

### 3.4 Expression of mRNAs associated with lipogenesis in the livers of MF and MSI‐1436 treated juvenile mice

Furthermore, qPCR also revealed that *Cd36* and *Acc* were significantly downregulated in the MSI‐1436‐treated livers, but unchanged in the MF‐treated animals (Figure 3). Specifically, *Cd36* was downregulated in MSI‐1436‐treated male mice compared with CTL‐ and MF‐treated male mice, with MSI‐1436‐treated male mice exhibiting less expression than MSI‐1436‐treated female mice attributed to an overall significant effect of treatment (*p* = 0.001) and sex (*p* = 0.02) (Figure 3a; Table S2). In addition, *Acc* was repressed in both MSI‐1436‐treated male and female mice relative to CTL‐ and MF‐treated female mice, with overall significant effect of treatment observed (*p* < 0.001) (Figure 3b). Interestingly, *Scd1* was significantly upregulated in the CTL female animals relative to the CTL male animals (*p* = 0.01), with significant effect of sex (*p* = 0.012) and interaction between sex and treatment noted (*p* = 0.046) (Figure 3c; Table S2).

### 3.5 Expression of mRNAs associated with energy sensing and fatty acid oxidation in the livers of MF and MSI‐1436 treated juvenile mice

To further explore the effects of MF and MSI‐1436 on liver physiology, we assessed the expression of *Sirt1* and *Cpt1* (Figure 3d,e). Downregulation in *Sirt1* expression was noted in the CTL‐ and MF‐treated female animals in comparison with the CTL‐ and MF‐treated male animals, suggesting a trend influenced by sex (*p* = 0.056) (Figure 3d; Table S2). Alongside, *Sirt1* levels were significantly diminished in both the MSI‐1436‐treated male and female livers relative to the CTL‐ and MF‐treated male and female groups. Multi‐factor ANOVA demonstrates both a significant effect of treatment (*p* < 0.001) and a noteworthy interaction between sex and treatment (*p* = 0.042) (Figure 3d). Similarly, relative to CTL‐ and MF‐treated male and female mice, we found that *Cpt1* was downregulated in the MSI‐1436‐treated male and female livers, with significant treatment effects observed (*p* < 0.001) (Figure 3e). In contrast, MF did not yield any notable changes in the expression of *Cpt1* (Figure 3e).

### 3.6 Expression of apolipoproteins in the livers of MF and MSI‐1436 treated juvenile mice

Next, we investigated the expression of lipid transport apolipoproteins *Apob*, *Apoe*, *Apoa1*, and *Apoa4* in the liver (Figure 4). Our analysis revealed that both MSI‐1436‐treated male and female mice demonstrated lower levels of *Apob* relative to CTL‐ and MF‐treated male mice, with overall significant effects of sex (*p* = 0.019) and treatment (*p* < 0.001) noted (Figure 4a). Of note, male mice across all groups appear to have higher levels of *Apob* relative female mice (Figure 4a), although not statistically significant. In addition, we observed that *Apoe* was downregulated in the livers of male and female mice treated with MSI‐1436 relative to CTL‐ and MF‐treated male and female mice, with a significant effect of treatment noted (*p* < 0.001) (Figure 4b). Furthermore, *Apoa1* was downregulated in the livers of male and female MSI‐1436‐treated mice relative to CTL‐ and MF‐treated male and female mice, respectively, with multi‐factor ANOVA indicating an overall significant effect attributed to treatment (*p* < 0.001) (Figure 4c). Alongside, a trend was noted in male mice which appeared to express higher levels of *Apoa1* across all treatment groups, with overall significant sex differences evident (*p* < 0.033) (Figure 4c). Lastly, *Apoa4* was downregulated in the MSI‐1436‐treated male and female livers relative to the CTL‐ and MF‐treated male and female livers, with an overall significant of treatment observed (*p* = 0.001) (Figure 4d). Specifically, Tukey pairwise analysis further revealed that MSI‐1436 treatment significantly inhibited the expression of *Apoa4* in both male and female mice relative to the CTL female mice (*p* = 0.024 and 0.026, respectively) (Table S2). Overall, MF‐treated animals did not exhibit significant alterations in the expression of the apolipoproteins assessed.

### 3.7 Expression of mRNAs involved with VLDL‐transport in the livers of MF and MSI‐1436 treated juvenile mice

We assessed the expression of VLDL‐transport associated mRNAs *Cideb*, *Mttp*, *Sar1a*, *Sar1b*, *Sec22b*, and *Stx5a* (Figure 5). Interestingly, *Cideb* was not significantly altered across any of the treatment groups (Figure 5a). *Mttp* was downregulated in the MSI‐1436‐treated female mice relative to the CTL‐ and MF‐treated female mice (Figure 5b). *Mttp* was also downregulated in the MF‐ and MSI‐1436‐treated male mice relative to the CTL‐treated male mice, with overall significant effect of treatment observed (*p* < 0.001) (Figure 5b). *Sar1a* was downregulated in both MSI‐treated male and female mice relative to CTL‐ and MF‐treated male and female mice, respectively, with an overall significant effect of treatment noted (*p* < 0.001) (Figure 5c). Alongside, *Sar1b* was significantly downregulated in the livers of MSI‐1436‐treated male and female mice relative to CTL‐ and MF‐treated male and female mice, respectively, with an overall significant effect of treatment observed (*p* < 0.001) (Figure 5d). Relative to CTL and MF mice, MSI‐1436‐treated male and female mice as expressed significantly less *Sec22b* with an overall effect of treatment observed (*p* < 0.001) (Figure 5e). Lastly, *Stx5a* appeared to be downregulated in the MSI‐1436 male and female mice with significant effect of treatment noted (*p* < 0.001) (Figure 5f). Overall, MF‐treated animals did not exhibit significant changes in the expression of the VLDL‐transport associated mRNAs assessed.

### 3.8 Expression of mRNAs involved with cholesterol metabolism in the livers of MF and MSI‐1436 treated juvenile mice

Lastly, to elucidate the impact of MF and MSI‐1436 treatment on liver function, we examined the expression of cholesterol metabolism associated mRNAs *Lxr*, *Abcg5*, and *Cyp7a1* (Figure S1). Particularly, *Lxr* was significantly downregulated in both the MSI‐treated male and female livers compared with the CTL male and female and MF‐treated male and female livers, respectively, with an overall significant effect due to treatment (*p* < 0.001) (Figure S1a). *Abcg5* was downregulated in the MSI‐1436‐treated male livers relative to the MF‐treated male livers with multi‐factor ANOVA indicating an overall significant effect attributed to sex (*p* = 0.002) and treatment (*p* = 0.010) (Figure S1b). *Cyp7a1* had a decreasing trend in the MF and MSI‐1436‐treated male and female livers relative to CTL male and female livers, respectively, with multi‐factor ANOVA indicating an overall significant effect due to treatment (*p* = 0.016) (Figure S1c). Overall, MF did not impart significant changes in the expression of the cholesterol transport associated mRNAs assessed.

### 3.9 MSI‐1436 treatment alters mRNA signatures in the liver regardless of sex

**FIGURE 6:**
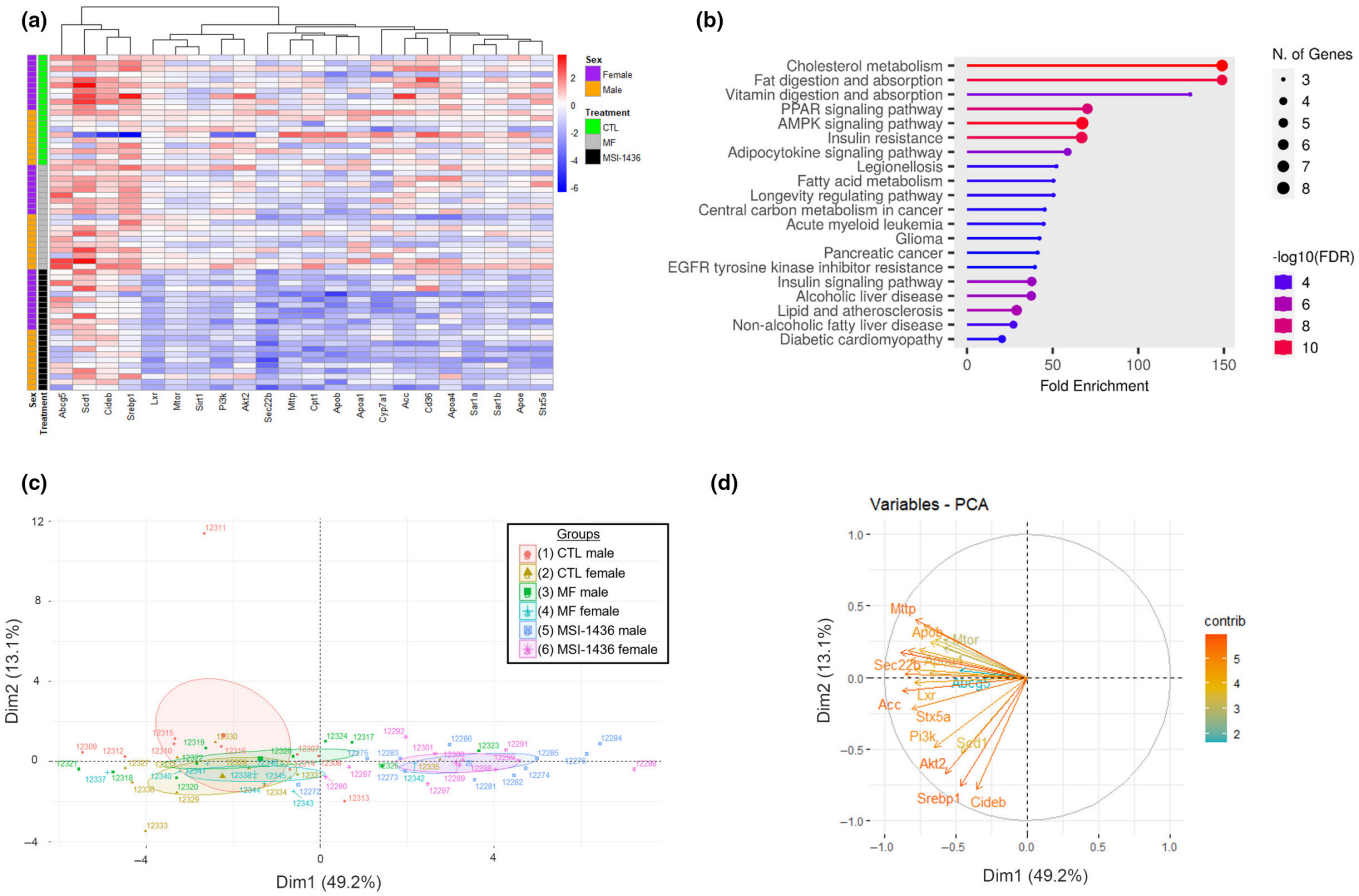
Gene expression analysis of mRNAs involved in metabolic processes in the livers of male and female UM‐HET3 mice. (a) Heatmap showing the overall expression profile of the 22 genes in the samples grouped by sex and treatment. Colors in the heatmap represent the value of −ΔΔCt ranging from downregulated (blue) to upregulated (red). (b) Gene enrichment analysis conducted using the ShinyGO 0.77 webtool with FDR cutoff of <0.05 to demonstrate the KEGG pathway associated with the mRNAs investigated. (c) PCA demonstrated that factor 1 explained 49.2% of variation mainly caused by different treatments, and that factor 2 explained 13.1% of variation mainly due to different sexes, and that the effect of MSI‐1436 treatment is similar for both sexes. −ΔΔCt method was used for gene expression calculation and normalization. Group 1: untreated male; Group 2: untreated female; Group 3: MF‐treated male; Group 4: MF‐treated female; Group 5: MSI‐1436‐treated male; and Group 6: MSI‐1436‐treated female. (d) PCA biplot showing the contribution of each target gene on the two major principal components. −ΔΔCt method was used for gene expression calculation and normalization.

In summary, both male and female MSI‐1436‐treated mice exhibited notable suppression of mRNAs involved with liver metabolic processes compared with CTL‐ and MF‐treated male and female mice (Figure 6a). These significantly downregulated mRNAs predominantly regulate pathways such as cholesterol metabolism, fat digestion and absorption, PPAR signaling, AMPK signaling, vitamin digestion and absorption, fatty acid metabolism, longevity, NAFLD, and insulin resistance (Figure 6b).

Alongside, principal component analysis (PCA) further illuminated that MSI‐1436 exerts a potent effect on the hepatic profiles of both male and female mice, evident by a discernible shift to the right along the x‐axis of Groups 5 and 6, distinctly separated and not overlapping with CTL (Groups 1 and 2) and MF‐treated (Groups 3 and 4) mice (Figure 6c). In this plot, dimension 1 (Dim1) represents the effect of treatment which contributes 49.2% of the observed variance, while dimension 2 (Dim2), reflecting sex differences, encompasses merely 13.1% of the observed variance, exhibiting that the effect of MSI‐1436 is similar for both sexes. Alongside, PCA also demonstrates near‐complete overlap between CTL‐ (Groups 1 and 2) and MF‐treated (Groups 3 and 4) male and female mice. Lastly, PCA biplot revealed the effect of each gene on the principal component dimensions, further demonstrating that the change in effects observed is largely attributed to the treatment rather than the sex of the animals (Figure 6d).

### 3.10 MF and MSI‐1436 influence the expression of miRNAs in the livers of male and female juvenile mice

**FIGURE 7:**
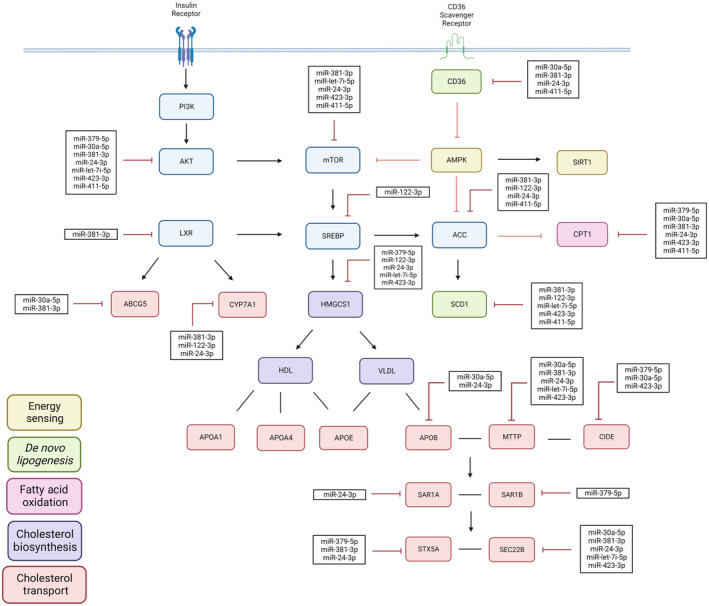
MSI‐1436 alters the expression of miRNAs and mRNAs to influence lipid metabolism associated signal transduction pathways in the livers of male and female UM‐HET3 mice. miRNA targets were predicted using the miRPathDB 2.0 bioinformatic webtool and previous literature findings. Pathways are separated by color; blue indicates involvement with multiple signal transduction pathways. Overall, in comparison with CTL and MF mice, MSI‐1436 significantly inhibits the expression of mRNAs involved with metabolic pathways in the liver. Created with BioRender.com.

We prepared miRNA sequencing libraries and investigated the significantly differentially expressed miRNAs in the livers of these animals. Using the ClustVis webtool, we generated heatmaps to assess whether MF or MSI‐1436 induce changes in the expression of miRNAs in the livers of young UM‐HET3 mice (Figure S2, Tables S3 and S4). We observed that both treatments influence the differential expression of miRNAs sex‐specifically (Figure S2a, Table S3). Due to the inherent biological sex differences observed in the CTL animals, samples were separated by sex to assess the treatment‐specific effects of the MF‐treated male and female mice against CTL male and female mice, respectively. Alongside, the treatment‐specific effects of MSI‐1436‐treated male and female mice were compared with CTL‐ and MF‐treated male and female mice, respectively (Figure S2b, Table S4). Significantly upregulated miRNAs and their respective predicted mRNA targets are illustrated in Figure 7.

### 3.11 Juvenile mice express distinct, sex‐specific miRNAs in the liver

Differential expression analysis revealed significant, biological sex‐specific differences in miRNA expression between the young UM‐HET3 male and female animals. Particularly, CTL male mice exhibited significant upregulation of miR‐1948‐3p, miR‐1948‐5p, miR‐122‐3p, and miR‐455‐3p and significant downregulation of miR‐802‐3p, miR‐378a‐5p, miR‐802‐5p, miR‐34a‐5p, and miR‐107‐3p relative to CTL female mice (Table 1). Functional analysis revealed that these sex‐specific miRNAs are involved in metabolic pathways, thyroid hormone signaling, *Pi3K‐Akt* signaling, *Ampk* signaling, insulin resistance, growth hormone synthesis and secretion, and *Foxo* signaling (Table S6).

**TABLE 1:** Significantly differentially expressed sex‐specific miRNAs.

Group	miRNA	*p*‐Value	FDR	LogFC
CTL M against CTL F	mmu‐miR‐1948‐3p	1.43E‐13	4.14E‐11	3.58862
	mmu‐miR‐455‐3p	5.28E‐06	0.000511	1.50293
	mmu‐miR‐122‐3p	0.000703	0.029276	1.44845
	mmu‐miR‐1948‐5p	0.001486	0.047888	1.89444
	mmu‐miR‐802–5p	1.01E‐10	1.47E‐08	−2.49255
	mmu‐miR‐802‐3p	2.96E‐05	0.002146	−4.13283
	mmu‐miR‐107‐3p	0.000382	0.022177	−1.0675
	mmu‐miR‐34a‐5p	0.000707	0.029276	−1.77335
	mmu‐miR‐378a‐5p	0.000982	0.035596	−1.41276
MF M against MF F	mmu‐miR‐1948‐3p	1.18E‐19	3.41E‐17	3.47153
	mmu‐miR‐194‐2‐3p	5.14E‐12	7.45E‐10	2.142
	mmu‐miR‐25‐3p	1.35E‐05	0.000975	1.01547
	mmu‐miR‐455‐3p	7.31E‐05	0.003426	1.77908
	mmu‐miR‐1948‐5p	0.000121	0.004393	1.56258
	mmu‐miR‐320‐3p	0.000602	0.014118	1.12821
	mmu‐miR‐140‐3p	0.000906	0.017519	0.92504
	mmu‐miR‐532–5p	0.001015	0.018394	0.86103
	mmu‐miR‐151‐3p	0.001338	0.021549	0.83811
	mmu‐miR‐28a‐3p	0.001951	0.029773	0.87756
	mmu‐miR‐351‐5p	0.002376	0.033226	1.12555
	mmu‐miR‐92a‐3p	0.002458	0.033226	0.7237
	mmu‐miR‐30e‐3p	0.002864	0.033226	0.91279
	mmu‐miR‐30a‐3p	0.003059	0.034115	0.89423
	mmu‐miR‐6239	0.00357	0.036971	0.88368
	mmu‐miR‐106b‐3p	0.004037	0.039026	1.21975
	mmu‐miR‐802–5p	7.14E‐09	6.9E‐07	−2.30543
	mmu‐miR‐32–5p	2.2E‐05	0.001276	−1.3473
	mmu‐miR‐106b‐5p	8.27E‐05	0.003426	−0.97622
	mmu‐miR‐126a‐5p	0.000312	0.010054	−0.98838
	mmu‐miR‐34a‐5p	0.000443	0.012835	−2.26052
	mmu‐miR‐142a‐3p	0.000504	0.013293	−1.38935
	mmu‐miR‐107‐3p	0.000633	0.014118	−0.86519
	mmu‐miR‐144‐3p	0.000756	0.015666	−1.08988
	mmu‐miR‐29b‐3p	0.00128	0.021549	−1.11874
	mmu‐miR‐802‐3p	0.002533	0.033226	−3.08912
	mmu‐miR‐1a‐3p	0.002807	0.033226	−2.66585
	mmu‐miR‐181c‐5p	0.002818	0.033226	−1.53809
	mmu‐miR‐29c‐3p	0.003255	0.034961	−0.75925
	mmu‐miR‐467a‐5p	0.003905	0.039026	−4.25435
	mmu‐miR‐192–5p	0.004251	0.039764	−0.60469
	mmu‐miR‐223‐3p	0.004603	0.040852	−0.88936
	mmu‐miR‐342‐3p	0.004649	0.040852	−2.43989
MSI‐1436 M against MSI‐1436 F	mmu‐miR‐802–5p	1.27E‐07	3.69E‐05	−1.7799

*Note*: All the significantly differentially expressed sex‐specific miRNAs between male (M) and female (F) control (CTL), metformin (MF), and trodusquemine (MSI‐1436) treated UM‐HET3 mice with respective *p*‐values and false discovery rates (FDRs) <0.05, alongside log fold changes (logFC).

### 3.12 MF and MSI‐1436 exert sex‐specific effects on liver miRNAs in male and female juvenile mice

Apart from miR‐122‐3p and miR‐378‐5p, both CTL‐ and MF‐treated male mice exhibited significant upregulation and downregulation of the same miRNAs relative to CTL‐ and MF‐treated female mice, respectively (Table 1). However, treatment with MF also appeared to induce and inhibit several new miRNAs that were not originally differentially expressed in the CTL animals, with functional analysis indicating their role in regulating processes including metabolism, insulin signaling, longevity, *Pi3K‐Akt* signaling, growth hormone signaling, *Mtor* signaling, hippo signaling, gonadotropin‐releasing hormone (GnRH) signaling, estrogen signaling, and thyroid hormone signaling (Table S6).

Apart from significant downregulation of miR‐802‐5p, miRNA profiling revealed that all the differentially expressed miRNAs between CTL male and female animals disappeared after treatment with MSI‐1436 (Table 1). Functional analysis revealed that mir‐802‐5p is involved with regulating cholesterol metabolism and fat digestion and absorption (Table S6).

### 3.13 MSI‐1436 exerts treatment‐specific effects on liver miRNAs in male and female juvenile mice

Although some treatment‐specific alterations were noted in the expression of miRNAs in the livers of the MF‐treated animals, these changes were not significant, with FDRs > 0.05 (Table S5). MSI‐1436 exerted treatment‐specific effects in both male and female mice against the CTL male and female groups, with *p*‐values and FDRs < 0.05 (Table 2). MSI‐1436 female mice demonstrated significant upregulation of miR‐379‐5p, miR‐30a‐3p, miR‐381‐3p, and miR‐122‐3p against CTL female mice (Table 2). MSI‐1436‐treated female mice were also characterized by significant downregulation of miR‐5099, miR‐6240, miR‐19b‐3p, miR‐6238, miR‐671‐5p, miR‐34a‐5p, miR‐378a‐5p, and miR‐6239 against CTL female mice (Table 2). MSI‐1436‐treated male mice displayed significant upregulation of miR‐379‐5p, miR‐24‐3p, miR‐let‐7i‐5p, and miR‐423‐3p against CTL male mice (Table 2). Additionally, MSI‐1436‐treated male mice also expressed significant downregulation of miR‐19b‐3p, miR‐100‐5p, miR‐144‐3p, miR‐365‐3p, miR‐101a‐3p, miR‐451a, and miR‐30b‐5p against CTL male mice (Table 2).

**TABLE 2:** Significantly differentially expressed treatment‐specific miRNAs.

Group	miRNA	*p* Value	FDR	LogFC
MSI‐1436 F against CTL F	mmu‐miR‐379‐5p	3.36E‐07	9.75E‐05	2.443510434
	mmu‐miR‐30a‐5p	0.000422	0.0185	1.064839426
	mmu‐miR‐381‐3p	0.001326	0.036664	4.107828295
	mmu‐miR‐122‐3p	0.001391	0.036664	1.332714594
	mmu‐miR‐5099	7.79E‐06	0.001129	−1.625841703
	mmu‐miR‐6240	4.59E‐05	0.004434	−2.599072436
	mmu‐miR‐19b‐3p	0.000167	0.012078	−1.704723387
	mmu‐miR‐6238	0.000344	0.0185	−2.24442914
	mmu‐miR‐671‐5p	0.000447	0.0185	−2.440717913
	mmu‐miR‐34a‐5p	0.000626	0.022695	−2.610880877
	mmu‐miR‐378a‐5p	0.001176	0.036664	−1.775341581
	mmu‐miR‐6239	0.001895	0.04579	−1.7264765
MSI‐1436 M against CTL M	mmu‐miR‐379‐5p	2.35E‐06	0.000341	2.050883293
	mmu‐miR‐24‐3p	7.72E‐06	0.000746	1.208948425
	mmu‐let‐7i‐5p	7.06E‐05	0.004097	0.865057809
	mmu‐miR‐423‐3p	0.000186	0.009007	1.062994349
	mmu‐miR‐19b‐3p	6.11E‐07	0.000177	−1.562474211
	mmu‐miR‐100‐5p	1.7E‐05	0.001233	−1.247054403
	mmu‐miR‐144‐3p	0.000428	0.016392	−1.428261313
	mmu‐miR‐365‐3p	0.000481	0.016392	−1.091905808
	mmu‐miR‐101a‐3p	0.000509	0.016392	−0.84283476
	mmu‐miR‐451a	0.000839	0.024327	−1.224504331
	mmu‐miR‐30b‐5p	0.001291	0.034028	−0.817858213
MSI‐1436 F against MF F	mmu‐miR‐30a‐5p	3.66E‐05	0.010621	1.061824394
	mmu‐miR‐379‐5p	0.000155	0.011258	1.987022937
	mmu‐miR‐5099	7.44E‐05	0.010784	−2.138929242
	mmu‐miR‐126a‐5p	0.00012	0.011258	−1.101267915
	mmu‐miR‐26b‐5p	0.000242	0.014008	−0.817421706
	mmu‐miR‐34a‐5p	0.000634	0.030623	−2.273277584
MSI‐1436 M against MF M	mmu‐miR‐379‐5p	6.12E‐06	0.001603	2.063183349
	mmu‐miR‐411‐5p	1.11E‐05	0.001603	1.924987013

*Note*: All the significantly differentially expressed treatment‐specific miRNAs between male (M) and female (F) control (CTL), metformin (MF), and trodusquemine (MSI‐1436) treated UM‐HET3 mice with respective *p*‐values and false discovery rates (FDRs) <0.05, alongside log fold changes (logFC).

Next, we compared the similarities and differences in miRNA differential expression patterns modulated by treatment with MSI‐1436. Regardless of sex, miR‐379‐5p was upregulated in both MSI‐1436‐treated female and male mice against CTL female and male mice with the highest log fold‐change, and miR‐19b‐3p was downregulated (Table 2). Crossover was also seen in expression of miR‐30a‐5p which was upregulated in MSI‐1436 female mice against CTL mice (Table 2). The same pattern was seen for miR‐5099 and miR‐34a‐5p which were downregulated in MSI‐1436‐treated female mice against CTL‐treated female mice (Table 2). In addition, MSI‐1436‐treated female mice exhibited downregulation of miR‐6240, miR‐6238, miR‐671‐5p, and miR‐378a‐5p against CTL female mice (Table 2). In comparison with CTL male mice, MSI‐1436‐treated male mice displayed downregulation of miR‐144‐3p and miR‐451a (Table 2).

### 3.14 Functional analysis of differentially expressed treatment‐specific miRNAs reveals that MSI‐1436 modulates development and metabolism in the livers of male and female juvenile mice

Pathway analysis using DIANA tools indicated that several important developmental and metabolic associated pathways are modulated by changes in miRNA expression influenced by treatment with MSI‐1436. Overall, MSI‐1436 appeared to modulate key pathways including growth hormone synthesis, secretion, and action, *Pi3K‐Akt* signaling, *Ampk* signaling, *Foxo* signaling, *Mtor* signaling, longevity regulating pathways, insulin signaling, insulin resistance, estrogen signaling, fatty acid biosynthesis, fatty acid metabolism, protein processing in the endoplasmic reticulum, and endocytosis (Table S7).

## 4 DISCUSSION

The postnatal period is a critical juncture in an organism's lifespan which governs a multitude of biological and physiological outcomes including growth, sexual maturation, reproductive capacity, disease susceptibility and pathogenesis, and overall survival (Bartke et al., 2022; Cameron & Demerath, 2002; Dorn et al., 2019; Kappeler et al., 2009). Particularly, the effects of growth hormone injections, food restriction, impaired ghrelin action, manipulation of litter size, and odor priming have all been examined in mouse pups during this vital developmental phase (Bartke et al., 2022; Garratt et al., 2022; Parra‐Vargas et al., 2023; Steculorum et al., 2015; Sun et al., 2009). These investigations underscore how early life intervention strategies can offer a window of opportunity to shape the adult phenotype thereby exerting long‐lasting impacts on evolutionary fitness. Targeting fundamental biomolecular pathways during this bout can reprogram metabolic and endocrine functions, yielding prospective benefits that could extend lifespan while concurrently improving the quality of life. As such, elucidating the effects of pharmaceutical agents such as MF and MSI‐1436 in young, developing mice can provide insight on whether these drugs can be utilized as novel methods to manage and prevent the onset of age‐related diseases, particularly those related to liver dysfunction.

Although many classes of drugs have already been pioneered to treat T2DM, diabetes management has steadily declined over the past decade, with fewer patients attaining glycemic control, blood pressure control, and cholesterol control (Fang et al., 2021). Current projections forecast a substantial rise in the prevalence of T2DM among adolescents, with a predicted increase of 700% (Xinhua, 2022). Particularly, early onset of T2DM exposes young individuals to chronic, long‐term hyperglycemia, which is associated with greater risk of mortality due to predisposition to many concomitant age‐related manifestations including cardiovascular disease, nonalcoholic fatty liver disease (NAFLD), and cancer presenting prematurely (Constantino et al., 2013; Morton et al., 2022). These alarming trends demonstrate the desperate need for novel pharmacotherapies that not only manage blood glucose levels, but also that can curb the pathogenesis of diabetes‐related comorbidities to improve the quality of life.

Over 70% of T2DM patients are also affected by NAFLD (Dharmalingam & Yamasandhi, 2018). To date, NAFLD is considered idiopathic with no standard treatment of care and inadequate patient compliance to lifestyle modifications, with only 50% of patients adhering to weight reduction protocols (Patanwala et al., 2021). Metformin (1,1‐dimethylbiguanide) is the first line of therapy in insulin‐resistant diabetes due to its ability to prevent hepatic gluconeogenesis, increase peripheral glucose uptake, and improve insulin sensitivity (Rena et al., 2017). Unfortunately, previous large‐scale studies of youth‐onset T2DM demonstrate that MF alone is ineffective at modulating glycemic control over time due to its diminished durability, suggesting the increased severity of the disease when diagnosed earlier in life (TODAY Study Group, 2012, 2021; Tryggestad & Willi, 2015). Alongside, MF treatment in obese children with T2DM has been shown to be unsuccessful at improving markers of NAFLD (Beauchamp et al., 2022). The burgeoning obesity epidemic has prompted a search for different pharmacological agents to target various mechanisms that are implicated in the pathogenesis of the disease in efforts to improve overall patient health outcomes and better clinical performance. Specifically, the *Pi3k/Akt* pathway is dysregulated in T2DM and is also a promising target for NAFLD due to its regulation of downstream *Mtor* and *Srebpc*, which induce lipogenesis in the liver (Feng et al., 2022; Huang et al., 2018).

MF is an FDA‐approved antidiabetic drug that is commonly employed to treat T2DM due to its potent ability to inhibit hepatic gluconeogenesis and lipogenesis (LaMoia & Shulman, 2021; Madiraju et al., 2014). Recently, MF has garnered extensive interest from the scientific community for its putative geroprotective effects (Glossmann & Lutz, 2019; Khan et al., 2023; Martin‐Montalvo et al., 2013; Novelle et al., 2016). Specifically, clinical trials have shown that MF can delay menarche, decrease the incidence of age‐related diseases, and prevent cognitive decline, indicating its robust ability to increase life expectancy and reduce mortality (Al‐Kuraishy et al., 2015, 2020, 2023; Ibanez, et al., 2011b; Ingram et al., 2006; Zhang et al., 2022; Zhu et al., 2022). Alongside, MSI‐1436, a reversible inhibitor of *Ptp1b*, is an experimental drug candidate that has similarly been regarded as a powerful modulator of metabolic diseases in mammals, with Phase II clinical trials further demonstrating its efficacy in treating obesity and T2DM (Bourebaba, Serwotka‐Suszczak, Bourebaba, et al., 2023; Cho, 2013; Takahashi et al., 2004). Our study aimed to elucidate how early administration of these compounds during the postnatal period can reconfigure hepatic function, prospectively deterring the onset of age‐related maladies across an organism's lifespan.

Analysis of physiological parameters including liver weight, non‐fasting glucose, and ITT and GTT indicates that MSI‐1436 had a potent effect on the livers of these animals relative to MF treatment. Particularly, we observed that MSI‐1436 increased liver to body weight ratio and decreased non‐fasting glucose in both male and female animals. Alongside, GTT was improved in male animals. MF appeared to improve GTT in female animals but impair ITT in the male animals. Additionally, we observed that, relative to MF, MSI‐1436 significantly inhibited the expression of key mRNA transcripts that govern the processes of cell growth and metabolism (*Pi3k*, *Akt2*, *Mtor*, *Srebp1*), fatty acid uptake and lipogenesis (*Cd36*, *Acc*, *Scd1*), energy sensing and fatty acid oxidation (*Sirt1*, *Cpt1*), cholesterol transport and homeostasis (*Apob*, *Apoe*, *Apoa1*, *Apoa4*, *Lxr*, *Abcg5*, *Cyp7a1*), and VLDL‐transport (*Cideb*, *Mttp*, *Sar1a*, *Sar1b*, *Sec22b*, *Stx5a*) in the livers of juvenile UM‐HET3 male and female mice. Particularly, these mRNAs play an instrumental role in regulating intricate biomolecular networks that are involved with cholesterol and fatty acid metabolism, fat digestion and absorption, and nutrient and energy signaling mediated through *Ppar* and *Ampk*. Moreover, these processes are integral to pathologies such as insulin resistance and nonalcoholic fatty liver disease (NAFLD), carrying implications for longevity and healthy aging. The observed changes underscore the exceptional sensitivity of the postnatal mouse liver to MSI‐1436, showcasing its robust ability to recalibrate metabolic functions and modify nutrient availability. These findings are of paramount importance as energy alterations during this critical developmental phase have far‐reaching effects beyond maintenance of liver health, profoundly influencing an organism's growth, determining the well‐being of extrahepatic organs, and consequently sculpting lifespan and quality of life (Patel & Srinivasan, 2010).

Although recent studies have illustrated that MF delays sexual maturation in 8‐week‐old female UM‐HET3 mice, evidenced by altered food consumption, tail‐length, vaginal patency, and metabolic traits, these changes may be attributed to mechanisms independent of *Pi3k*, *Akt2*, *Mtor*, and their downstream pathways (Zhu et al., 2022). Our study demonstrates that MF had a limited effect on mRNA transcripts conventionally responsible for driving fundamental metabolic processes in the liver. Specifically, this suggests that alternate molecular networks may be at play to contribute to the phenotypic changes noted by MF treatment in previous studies, with factors such as hormonal variations and interactions with the gut microbiota potentially orchestrating the perceived differences (Khan et al., 2023; Vallianou et al., 2019). Furthermore, the dosage and timing of MF administration could be crucial determinants of the study outcomes observed. Therefore, it is essential for future studies to modify the treatment regiment, identify alternate targets, measure blood hormone levels, assess the relative abundance of bacterial populations, and consider extending the duration of the study to dissect the underlying mechanisms which could contribute to delayed growth and sexual maturation, thereby extending lifespan.

To further investigate the complex, multifaceted biomolecular impacts of MF and MSI‐1436 treatments in young, postnatal mice, we assessed the differential expression patterns of liver‐specific miRNAs. miRNAs are small (~22 nucleotide), noncoding RNAs that possess the unique ability to regulate a wide array of biomolecular functions by selectively binding to complementary mRNA sequences, subsequently prompting mRNA degradation and repressing translation (Cai et al., 2009; O'Brien et al., 2018). As such, miRNA signatures are regarded as valuable diagnostic and prognostic tools with promising therapeutic applications. By conducting miRNA profiling in response to MF and MSI‐1436, we aimed to shed light on how these therapeutic agents can influence potent biomolecular switches to shape an organism's developmental and metabolic landscape.

In our study, we uncovered a distinct pattern of miRNAs between male and female CTL UM‐HET3 mice, underscoring the pivotal role that biological sex plays in shaping both developmental and metabolic trajectories in the liver. Particularly, our data revealed that differentially expressed miRNAs in male UM‐HET3 mice may regulate growth hormone and thyroid hormone signaling, cholesterol metabolism, PPAR signaling, insulin resistance, and longevity differently in comparison with their female counterparts. Additionally, we found that only miR‐122‐3p and miR‐378a‐5p were absent in the male animals after MF treatment whereas all other differentially expressed miRNAs altered between CTL male and female animals remained. This observation suggests that MF has a minimal effect on changing liver metabolic processes distinctly regulated by sex, further indicating its age‐dependent or dose‐dependent effects (TODAY Study Group, 2012).

Particularly, CTL‐ and MF‐treated male mice exhibited upregulation of miR‐1948‐3p, miR‐1948‐5p, and miR‐455‐5p and downregulation of miR‐802‐3p, miR‐107‐3p, and miR‐34a‐5p. Previous studies have shown that miR‐1948 and miR‐802 are expressed sex‐specifically due to their role in postnatal liver development, with miR‐1948 upregulated and miR802 downregulated in male mice between 4 and 8 weeks of age (Hao & Waxman, 2018). In addition, research has demonstrated that miR‐802 is induced in obese mice due to its inhibition of *Ampk*, the direct target of MF, resulting in the development of NAFLD (Sun et al., 2022). Furthermore, miR‐34a‐5p was also significantly downregulated in both the CTL‐ and MF‐treated male livers. miR‐34‐a is a known biomarker for NAFLD and has been shown to dysregulate energy sensing by inactivating *Ampk* and *Sirt1* expression in the liver (Ding et al., 2015). While we noted significant downregulation of this miRNA, we did not observe a corresponding significant increase in *Sirt1*, *Ampk*, or downstream *Cpt1* in MF‐treated animals against the CTL demonstrating its inadequacy at modulating liver homeostasis at this dosing regimen in young animals (TODAY Study Group, 2012).

Furthermore, MF also appeared to induce new miRNAs not originally present in the CTL male and female animals, with pathway analysis demonstrating that these miRNAs may be involved with growth hormone, estrogen, and thyroid hormone signaling which may have implications in postnatal development and aging. Surprisingly, although miR‐455 was upregulated in MF‐treated male mice, there was no effect on targets *Srebp*, *Fas*, *Scd1*, and *Acc* which are typically downregulated by its induction (Fang et al., 2020). Furthermore, the downregulation of miR‐107‐3p has been shown to decrease the expression of *Srebp* and increase the expression of *Cpt1* (Gracia et al., 2017). However, while we observed downregulation of miR‐107‐3p in MF‐treated male animals, we did not see a significant decrease of *Srebp* or a significant increase of *Cpt1*. Lastly, miR‐122‐3p was upregulated and miR‐378‐5p was downregulated, both being uniquely expressed in the CTL male livers relative to female livers. miR‐122‐3p has been shown to prevent metabolic diseases by inhibiting *Fas*, *HmgCoA*, and *Srebp* and also has implications in steroid hormone biosynthesis (Alisi et al., 2009; Tsai et al., 2012). Overall, CTL‐ and MF‐treated male mice demonstrate sex‐specific differences in the expression of miRNAs and mRNAs that are involved with the pathogenesis of metabolic diseases. Additionally, MF treatment resulted in differential expression of new miRNAs that were not originally present in CTL male animals. The role of these miRNAs is unknown and needs to be elucidated in the context of postnatal liver development regarding its impact on health and lifespan.

In addition, we observed that apart from downregulation of miR‐802–5p, treatment with MSI‐1436 minimized the differences observed between CTL male animals and CTL female animals, demonstrating its ability to harmonize metabolic disparities imparted by biological sex. This may explain its potent effect on liver function or may indicate its role in mitigating sex‐specific differences to potentially delay development. Of note, previous studies have demonstrated that MSI‐1436 significantly represses the expression of miR‐802–5p in the equine liver, with our functional enrichment analysis proposing its involvement in modulating cholesterol metabolism and fat digestion and absorption (Bourebaba, Serwotka‐Suszczak, Bourebaba, et al., 2023). Coupled with our qPCR data, these findings indicate that MSI‐1436 significantly influences miRNAs and mRNAs involved with liver metabolism in a sex‐independent manner.

miR‐379‐5p was the only miRNA that was upregulated in the livers of both MSI‐1436‐treated male and female mice in comparison with both CTL‐treated male and female mice, respectively, suggesting its central role in regulating liver physiology. Studies have shown that the miR379 cluster is critical to neonatal liver energy homeostasis and that its deletion can be lethal (Labialle et al., 2014). Functional analysis further revealed that this miRNA is involved in modulating pathways including carbohydrate metabolism, glycolysis, and gluconeogenesis, with recent studies disclosing that it is significantly downregulated in the livers of patients with NAFLD as well as in mouse models of diabetes (Dong et al., 2022). Namely, the upregulation of miR‐379‐5p has been found to modulate cholesterol homeostasis and prevent hepatic lipotoxicity through targeting of the *Stat1/Hmgcs1* axis (Dong et al., 2022). These findings indicate that miR‐379‐5p is a robust regulator of liver health through modulation of cholesterol homeostasis. We also identified several downregulated miRNAs in response to MSI‐1436 in both male and female animals. Particularly, miR‐34a‐5p was found to be significantly downregulated in the MSI‐1436‐treated female mice relative to both CTL‐ and MF‐treated female mice. Several studies indicate that miR‐34a‐5p is upregulated in metabolic diseases due to its ability to induce fatty acid biosynthesis and inhibit fatty acid oxidation in the liver (Xu et al., 2021). Furthermore, miR‐19b‐3p was downregulated in both MSI‐1436‐treated male and female mice against CTL male and female mice, respectively, with literature indicating that its expression is decreased in the plasma of patients NAFLD, and its expression in the liver yet to be elucidated (Vulf et al., 2021).

Our findings further reveal that MSI‐1436 uniquely upregulates miR‐122‐3p in MSI‐1436‐treated female mice relative to CTL female mice. Importantly, literature analysis revealed that miR‐122‐3p is the most abundant miRNA in the liver and is responsible for directly inhibiting PTP1B, the direct target of MSI‐1436 (Tsai et al., 2012; Yang et al., 2012). Previous studies maintain that miR‐122‐3p acts as a key regulator of lipid and cholesterol metabolism through direct modulation of lipogenic genes such as *Fas*, *HmgCoa*, and *Srebp* (Tsai et al., 2012; Wu et al., 2017; Yang et al., 2012). Interestingly, miR‐122 expression is typically similarly expressed between male and female mice during postnatal development, between 4 and 8 weeks of age (Hao & Waxman, 2018). Additionally, we also observed that the MSI‐1436‐treated male animals were characterized by upregulation of miR‐24‐3p, miR‐let‐7i‐5p, miR‐423‐3p, and miR‐411‐5p. Literature search revealed that mesenchymal stem‐cell‐derived exosomal miR‐24‐3p prevents inflammation and lipogenesis in the liver through targeting of *Keap1* and *Sting* and that miR‐411‐5p levels are decreased in the serum exosomes and livers of patients with NAFLD and nonalcoholic steatohepatitis (NASH) (Du et al., 2022; Wan et al., 2022). However, in other studies these miRNAs have also been associated with the pathogenesis of NAFLD (Ng et al., 2014; Zobeiri et al., 2021). Interestingly, miR‐423‐3p has been reported to be increased in the plasma of prepubertal children with obesity (Santos et al., 2023). Further studies indicate that higher levels of miR‐423‐3p in the liver and plasma are associated with insulin resistance and NAFLD and that miR‐423‐5p induces gluconeogenesis and lipogenesis (Yang et al., 2017). Additionally, some studies indicate that overexpression of the miR‐let7 family can induce insulin resistance and glucose intolerance thereby contributing to the pathogenesis of T2DM (Frost & Olson, 2011; Simino et al., 2021). However, the expression miR‐let7 specifically in the liver and its role in NAFLD has not been well elucidated. Thus, the push–pull dynamics at play must be more thoroughly investigated with future studies necessary to assess the expression of miR‐423‐3p and miR‐let‐7i‐5p in the liver and to understand their role in regulating liver health.

Our study demonstrates that MF does not influence the liver metabolic processes that we investigated, indicating its age‐dependent and dose‐dependent effects, particularly evident by lack of induction of its direct target *Sirt1* in our assessment (TODAY Study Group, 2012). Perhaps, MF may be driving metabolic pathways in the liver through mediation of other signal transduction mechanisms in young mice, further substantiated by our qPCR analysis which revealed its nominal impact on the *Pi3k/Akt/Mtor* pathway (Guo et al., 2021; Zhou et al., 2001).

By investigating whether MSI‐1436 can delay growth, we may uncover novel early intervention strategies that can facilitate lifespan extension and slow the aging process to prevent the onset of metabolic diseases. Importantly, miR‐379‐5p, a known modulator of cholesterol metabolism, reported to be inhibited in the livers of patients with NAFLD and in mouse models of diabetes, was found to be significantly upregulated by MSI‐1436 in both sexes, suggesting its central role in maintaining liver health. Our study identifies novel targets for metabolic conditions and yields valuable insight on the therapeutic potential of MSI‐1436 in treating obesity and concurrent liver diseases in juvenile mice regardless of sex. As such, we conclude that MSI‐1436 may be more capable of treating concomitant illnesses such as fatty liver, making it a more viable option for individuals suffering from both T2DM and NAFLD. We believe that MSI‐1436 is a promising pharmacological treatment that could be administered to both men and women suffering from metabolic diseases to improve glycemic load, prevent insulin resistance, and minimize triglyceride retention to improve the quality of life. Our study highlights the mechanism by which MSI‐1436 regulates liver health and elucidates novel biomarkers that could be used as therapeutic targets to help ameliorate the burden of early onset T2DM and related comorbidities and to prevent premature death. Future studies are necessary to assess the impacts of MF on sex hormones and liver development mechanisms and of MSI‐1436 on plasma profiles to understand its effects on circulating triglyceride and cholesterol levels to corroborate the role of these miRNAs in disease models of T2DM and NAFLD.

In conclusion, we have discovered, in comparison with MF, MSI‐1436 more potently influences the expression of miRNAs and mRNAs involved with *Pi3k/Akt/Mtor* signaling and downstream processes of lipogenesis and cholesterol biosynthesis in the livers of male and female UM‐HET3 mice. The ability of MSI‐1436 to influence genes central to metabolism and energy balance is remarkable due to the potential implications on development and the long‐term ramifications on healthspan. These transcriptomic alterations may be attributed to its direct inhibition of *Ptp1b* which is a known modulator of insulin, leptin, GH, and *Igf1* signaling (Owen et al., 2015). Given the low dose of MSI‐1436 employed in the study and the subsequent disappearance of key miRNAs present in CTL animals that are involved with regulating postnatal development and metabolism, MSI‐1436 should be used cautiously as it exerts robust effects on liver homeostasis. Furthermore, research should also focus on how these drugs affect extrahepatic organs and processes and should further explore the physical parameters that are improved through administration of these compounds.

In summary, the postnatal period offers a unique window of opportunity for interventions aimed at mitigating the effects of aging and age‐related diseases. Pharmaceutical agents like MF and MSI‐1436 are excellent candidates for such early life intervention strategies. The key novel aspects of this study include exploration of the effects of early life treatment with MF and MSI‐1436 and direct comparison of their actions in the same stock of experimental animals. Importantly, this stock of mice was designed to have considerable genetic heterogeneity and thus resemble the genetic architecture of human populations. Therefore, experimental findings obtained in these animals are potentially more translatable to human use. While MF has been widely used for many years, MSI‐1436 has only been studied in early stage human clinical trials in adults (Smith et al., 2017). Clinical evaluation of both these compounds during the period of rapid prepubertal growth in humans remains to be explored.

In particular, previous studies have demonstrated that MF monotherapy in children with T2DM is only effective 50% of the time warranting the need for new or combination therapies such as MSI‐1436 which has demonstrated its ability to reduce cholesterol levels in both rodents and humans (Bourebaba, Serwotka‐Suszczak, Bourebaba, et al., 2023; Bourebaba, Serwotka‐Suszczak, Pielok, et al., 2023; TODAY Study Group, 2012, 2021; Lantz et al., 2010; Marcus et al., 2022; Smith et al., 2017; Tryggestad & Willi, 2015). Lastly, the ability of MF to delay menarche and protect against age‐related diseases showcases its potential as an anti‐aging drug (Ibanez, et al., 2011a; Zhu et al., 2022). Similarly, the protective effects of MSI‐1436 against neurodegenerative diseases and age‐related conditions highlight its geroprotective potential (Bourebaba, Serwotka‐Suszczak, Bourebaba, et al., 2023; Olloquequi et al., 2022). Future longitudinal studies are necessary to elucidate the long‐term impacts of administering MF and MSI‐1436 during the postnatal period, particularly through functional studies to establish whether they can extend lifespan and improve the quality of life.

## AUTHOR CONTRIBUTIONS

SAA, YZ, YF, AB, MZ, SS, and MMM conceptualized the project. SAA, AS, XS, MMM, YZ, YF, and AB developed the methodology. SAA, MAMM, BMZ, DNG, YZ YF, DM, SM, RS, and RY performed the experiments. SAA, AS, XS, YZ, YF, AB, MZ, and SS, MMM were responsible for visualization. AB, SS, and MMM acquired funding. AB and MMM supervised the project. SAA wrote the original draft of the manuscript. SAA, AS, XS, EJ, MAMM, BMZ, NH, AB, MZ, SS, and MMM were responsible for editing and review of the manuscript.

## FUNDING INFORMATION

This work was supported by the National Institute on Aging of the National Institutes of Health R56 AG074499 (MM), National Science Foundation Award Number (FAIN): 2317758, the American Diabetes Association grant ADA 1‐19‐IBS‐126 (AB), the National Institute on Aging R21 AG062985 (AB), and the National Institutes of Health RO1 DK‐125596 (SS).

## CONFLICT OF INTEREST STATEMENT

The authors declare that no conflict of interest exists.

## Supporting information


Appendix S1.

Appendix S2.

Appendix S3.

## Data Availability

The data that support the findings of this study are openly available in NCBI BioProject at https://www.ncbi.nlm.nih.gov/bioproject/1067997, reference number PRJNA1067997.
