# Supplementary material for: Early life interventions metformin and trodusquemine metabolically reprogram the developing mouse liver through transcriptomic alterations
Source: Aging Cell. 2024 May 27;23(9):e14227. doi: 10.1111/acel.14227 (PMC11488326; doi:10.1111/acel.14227)
Supplement: Supplementary file 2 — Appendix S2. [file ACEL-23-e14227-s001.zip › acel14227-sup-0002-AppendixS2.docx]

| Supplemental Table 1: *Mus musculus* Primer List. | | |  |
| --- | --- | --- | --- |
| **mRNA** | **Forward (5' 🡪 3')** | **Reverse (5' 🡪 3')** | |
| *β2M* | AAGTATACTCACGCCACCCA | CAGGCGTATGTATCAGTCTC | |
| *Pi3k* | TAGCTGCATTGGAGCTCCTT | TACGAACTGTGGGAGCAGAT | |
| *Akt2* | GAGGACCTTCCATGTAGACT | CTCAGATGTGGAAGAGTCAC | |
| *Mtor* | CGGCAACTTGACCATCCTCT | TGCTGGAAGGCGTCAATCTT | |
| *Srebp1* | AAGACATGCTCCAGCTCATC | TACGCACGTCGAACGAAACT | |
| *Cd36* | GCCCAATGGAGCCATCTTTG | AGCTGCTACA GCCAGATTCA | |
| *Acc* | GTCCCCAGGGATGAACCAATA | GCCATGCTCAACCAAAGTAGC | |
| *Scd1* | GAGGCCTGTACGGGATCATA | TGAGAGAAGAAGAAGCCACGG | |
| *Sirt1* | CAGTGAGAAAATGCTGGCCTA | TTGGTGGTACAAACAGGTATTGA | |
| *Cpt1* | CTCAGTGGGAGCGACTCTTCA | GGCCTCTGTGGTACACGACAA | |
| *Apob* | CGTGGGCTCCAGCATTCTA | TCACCAGTCATTTCTGCCTTTG | |
| *Apoe* | GCTGGGTGCAGACGCTTT | TGCCGTCAGTTCTTGTGTGACT | |
| *Apoa1* | CCACACCCTTCAGGATGAAAG | TGGCTCCCTGTCAGGAAGAC | |
| *Apoa4* | CAGCTGACCCCATACATCCAG | TCATCGAGGTGTGCAGGTTG | |
| *Cideb* | TCTTTGGCAGCCTCAACGTGA | TGAAGCAGCGATTACGGTGGG | |
| *Mttp* | CTCTTGGCAGTGCTTTTTCTCT | GAGCTTGTATAGCCGCTCATT | |
| *Sar1a* | GGGCCGTTGTAAGCATCAATA | TTCCAGATTTCTTGTAGAGTCCTAGGA | |
| *Sar1b* | GCTAAAAAGGCAGGGCTATGG | GGCCGCTGCTAATCGATGTA | |
| *Sec22b* | CATTGATAGCCGTGCTCGGAG | CGCATCTTCAAGTACTTCGCATC | |
| *Stx5a* | CGGAAACGCTACGGATCTAAG | CAGGGGACAGAACCTGTGT | |
| *Lxr* | TGCCATCAGCATCTTCTCTG | GGCTCACCAGCTTCATTAGC | |
| *Abcg5* | ATCCAACACCTCTATGCTAAATCAC | TACATTATTGGACCAGTTCAGTCAC | |
| *Cyp7a1* | CCAGGGAGATGCTCTGTGTTC | ACCCAGACAGCGCTCTTTGAT | |

List of mouse primers used to conduct qPCR in the livers of male and female UMHET3 mice. Manufactured through Integrated DNA Technologies.

Supplemental Table 5: Differentially expressed treatment-specific miRNAs in the livers of MF treated mice.

| Group | miRNA | P Value | FDR | logFC |
| --- | --- | --- | --- | --- |
| MF M against CTL M | mmu-miR-15a-5p | 0.00354 | 0.52769 | -0.8213 |
|  | mmu-miR-144-3p | 0.00367 | 0.52769 | -1.1692 |
|  | mmu-miR-29c-3p | 0.00546 | 0.52769 | -0.7025 |
|  | mmu-miR-451a | 0.00927 | 0.63105 | -0.9701 |
|  | mmu-miR-29b-3p | 0.01924 | 0.65805 | -0.905 |
|  | mmu-miR-99a-5p | 0.0298 | 0.71184 | -0.4799 |
|  | mmu-miR-106b-5p | 0.02991 | 0.71184 | -0.5803 |
|  | mmu-let-7i-3p | 0.03029 | 0.71184 | -1.1491 |
| MF F against CTL F | mmu-miR-1a-3p | 0.00113 | 0.327 | 2.98981 |
|  | mmu-miR-144-3p | 0.00261 | 0.3549 | 1.04962 |
|  | mmu-miR-32-5p | 0.00784 | 0.40207 | 0.73102 |
|  | mmu-miR-6240 | 0.01116 | 0.63105 | 1.07074 |
|  | mmu-miR-320-3p | 0.01306 | 0.63105 | 0.84996 |
|  | mmu-miR-222-3p | 0.01601 | 0.65805 | 0.62944 |
|  | mmu-miR-125a-5p | 0.02042 | 0.65805 | 0.88008 |
|  | mmu-miR-194-2-3p | 0.03191 | 0.71184 | 0.57938 |
|  | mmu-miR-429-3p | 0.04852 | 0.91827 | 1.51406 |
|  | mmu-miR-671-5p | 0.00367 | 0.3549 | -1.5542 |
|  | mmu-miR-19b-3p | 0.00576 | 0.40207 | -1.0443 |
|  | mmu-miR-500-3p | 0.00832 | 0.40207 | -1.9708 |
|  | mmu-miR-378a-3p | 0.01181 | 0.48934 | -1.0779 |
|  | mmu-miR-378c | 0.01695 | 0.61268 | -1.1374 |
|  | mmu-miR-193a-3p | 0.0197 | 0.61268 | -1.9964 |
|  | mmu-miR-511-3p | 0.02113 | 0.61268 | -1.1823 |
|  | mmu-miR-6240 | 0.02737 | 0.68206 | -1.1202 |
|  | mmu-miR-326-3p | 0.02822 | 0.68206 | -1.35 |
|  | mmu-miR-194-2-3p | 0.03514 | 0.70342 | -0.756 |
|  | mmu-miR-185-5p | 0.03561 | 0.70342 | -0.6086 |
|  | mmu-miR-6238 | 0.03849 | 0.70342 | -1.1356 |
|  | mmu-miR-874-3p | 0.0425 | 0.70342 | -1.1719 |
|  | mmu-miR-6236 | 0.04293 | 0.70342 | -0.9864 |
|  | mmu-miR-6538 | 0.04366 | 0.70342 | -1.5877 |
|  | mmu-miR-320-3p | 0.04776 | 0.72895 | -0.6803 |

All the differentially expressed upregulated and downregulated treatment-specific miRNAs of metformin (MF) treated male (M) and female (F) UMHET3 mice against control (CTL) male (M) and female (F) UMHET3 mice with p values < 0.05, while FDRs are >0.05.

Supplemental Table 8: Summary of findings in existing literature on MSI-1436. (PD: Parkinson’s disease; AD: Alzheimer’s disease; FTD: frontotemporal dementia; NASH: non-alcoholic steatohepatitis; NAFLD: non-alcoholic fatty liver disease)

| **Molecular Target** | **Effect on target cell/organ** | **Disease** |
| --- | --- | --- |
| Cellular membranes | Displacement of misfolded proteins (alpha-synuclein, beta amyloid, TDP-43) [112-115] | Aging related diseases, PD, AD |
| Cellular membranes | Inhibition of misfolded protein aggregation[112-115] | Aging related diseases, PD, AD, FTD |
| Cellular membranes | Increase in membrane strength, fluidity, lipid distribution[114, 116, 117] | Aging related diseases, PD, AD, FTD |
| Cellular membranes | Prevention of membrane damage by toxic protein aggregates[112-116, 118-121] | Aging related diseases, PD, AD, FTD |
| Unknown | RBM3 induction (synaptogenesis) (unpublished) | Cognitive impairment/AD |
| PTP1B | Reversal of inflammation, neuronal loss, inflammation, tau phosphorylation and extension of healthy lifespan in Tau AD model (unpublished) | Aging related diseases, PD, AD, FTD |
| PTP1B | Reversal of glial inflammation and neuronal loss in a beta amyloid AD model (HAPP-J20)[122] | Aging related diseases, PD, AD, FTD |
| PTP1B | Improved synaptic plasticity in hippocampus[123] | AD, PD, age-related cognitive impairment |
| PTP1B | Sensitization of Insulin receptor[44, 45, 49, 124-126] | AD, PD, age-related cognitive impairment, Type 2 Diabetes, obesity |
| PTP1B | Sensitization of Leptin Receptor[44, 56] | AD, PD, age-related cognitive impairment, Type 2 Diabetes, obesity |
| PTP1B | Stimulation of amygdalar endocannabinoid release[127, 128] | Anxiety, AD, PD |
| PTP1B | Mobilization of hepatic lipids[45, 56] | Fatty liver disease, NASH, NAFLD |
| PTP1B | Reduction in ER stress[45, 47, 129-131] | Aging related diseases |
| PTP1B | Improvement in mitochondrial function[47, 130-132] | Aging related diseases |
| PTP1B | Inhibits PTP1B oncogenic activity[133, 134] | Breast Cancer |
| PTP1B | Stimulates T cell anti-tumor immunity[134] | Aging related diseases, Cancer |
| PTP1B | Restores flow induced arteriole dilatation [129] | Cerebrovascular disease |
| PTP1B | Reverses pre-existing atheroma[135] | Cardiovascular disease and stroke |
| PTP1B | Prevents aortic valve calcification[132] | Valvular heart disease |
| PTP1B | Mobilizes cardiac stem cells post myocardial infarction and inhibits fibrotic repair[46] | Myocardial infarction |
| PTP1B | Mobilizes skeletal muscle stem cells following traumatic injury[46] | Sarcopenia, traumatic injury |


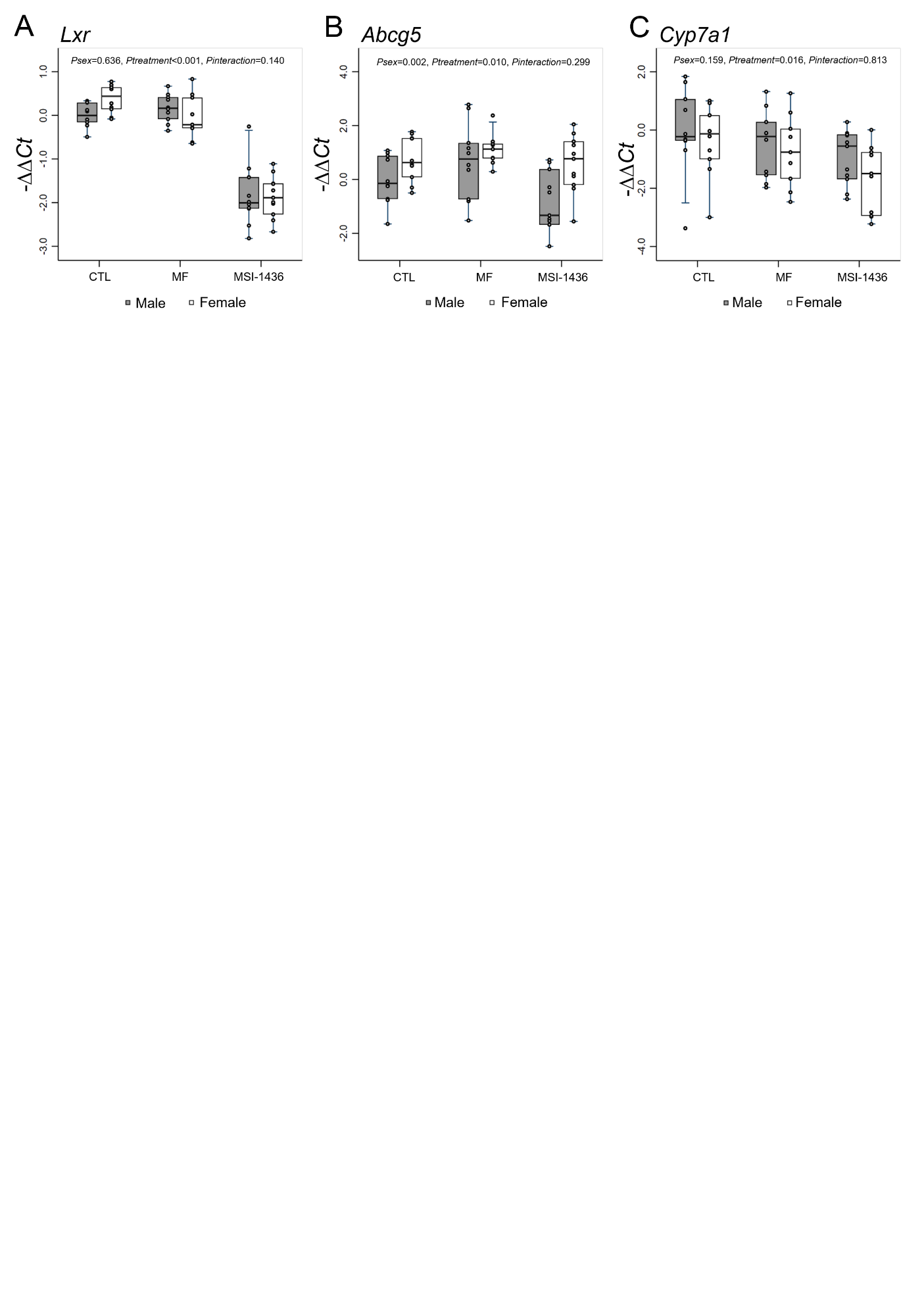


***Supplemental Figure 1. qPCR analysis of mRNAs Involved with Cholesterol Metabolism.*** -∆∆Ct method was used to calculate and normalize gene expression of each target gene, with β2M selected as a housekeeping gene and untreated male animals assigned as control samples. Stata MP15 and R packages were used to conduct statistical analysis and generate box-and-whisker plots. Multi-factor ANOVA was performed with interaction between treatment and sex included, and post-hoc analysis was conducted using Tukey’s multiple pairwise comparisons over sex and treatment.


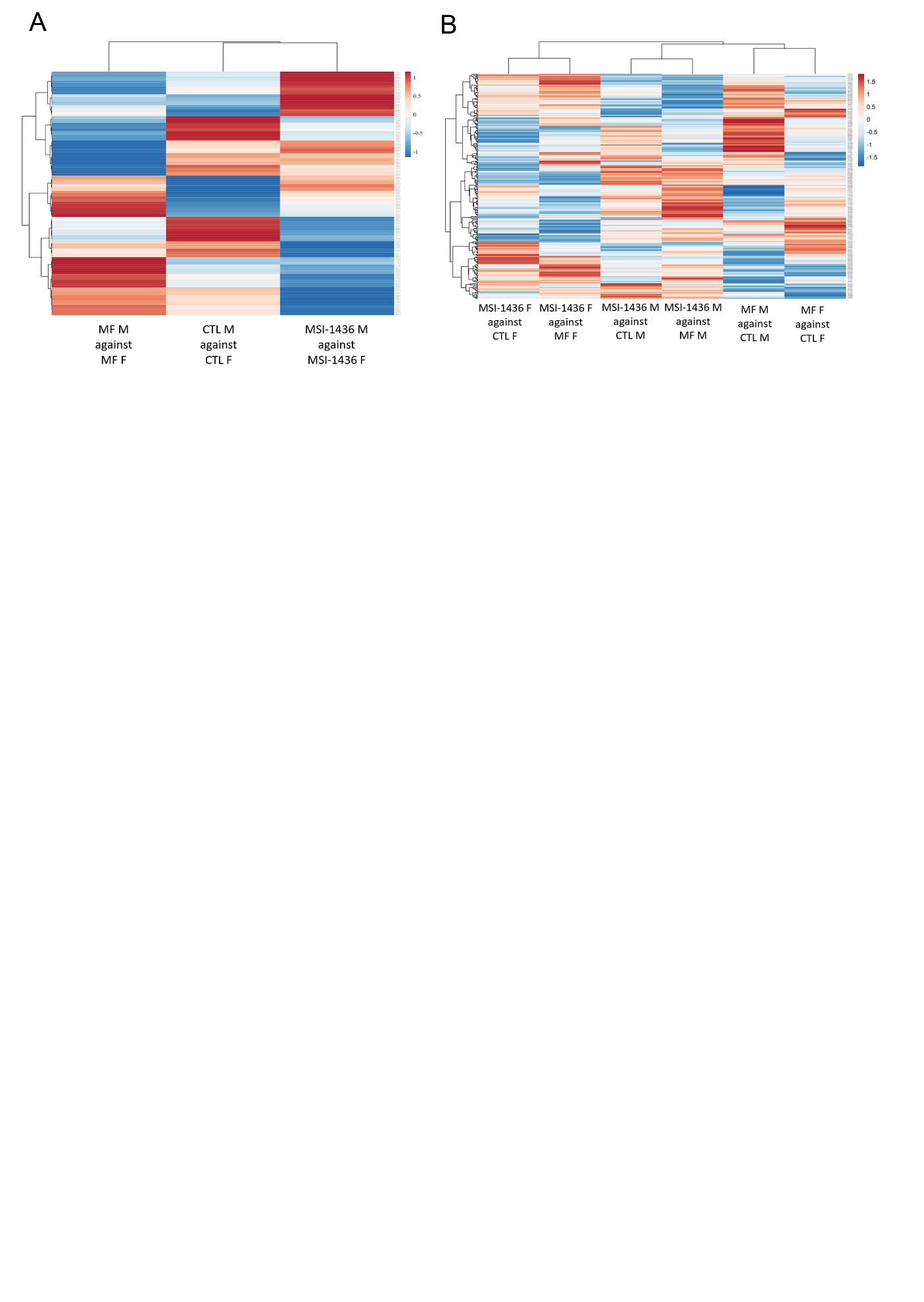


***Supplemental Figure 2. Heatmap of the Detected miRNAs in the Livers of CTL, MF, and MSI-1436 Treated Male and Female UMHET3 Mice Generated Using the Online ClustVis webtool.*** **(A)** Sex-Specific miRNA sequencing analysis from the livers of CTL, MF and MSI-1436 treated UMHET3 male (M) mice against CTL, MF, and MSI-1436 treated UMHET3 female (F) mice, respectively. **(B)** Treatment-specific miRNA sequencing analysis of MF and MSI-1436 treated UMHET3 male (M) and female (F) livers. Red color indicates FC>0 while blue color indicates FC<0. See PDF files of Supplemental Figures 2A and 2B and Supplemental Tables 3 and 4 for specifics regarding depicted y-axis further detailing the list of identified miRNAs and respective FCs.
