# Supplementary figures and images for: Early life interventions metformin and trodusquemine metabolically reprogram the developing mouse liver through transcriptomic alterations
Source: Aging Cell. 2024 May 27;23(9):e14227. doi: 10.1111/acel.14227 (PMC11488326; doi:10.1111/acel.14227)

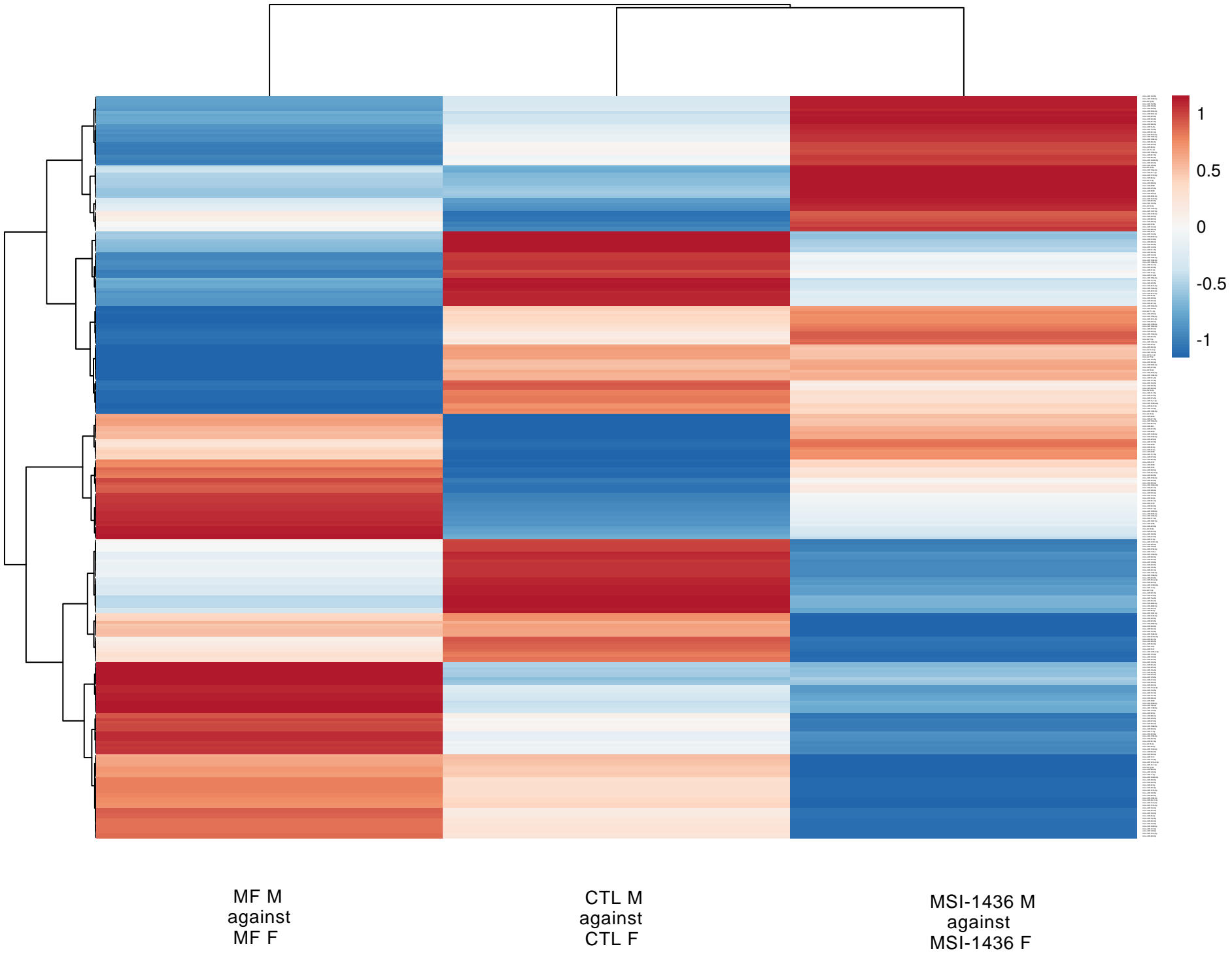

Supplement: Supplementary file 2 — Appendix S2. [file ACEL-23-e14227-s001.zip › acel14227-sup-0003-AppendixS3.pdf]

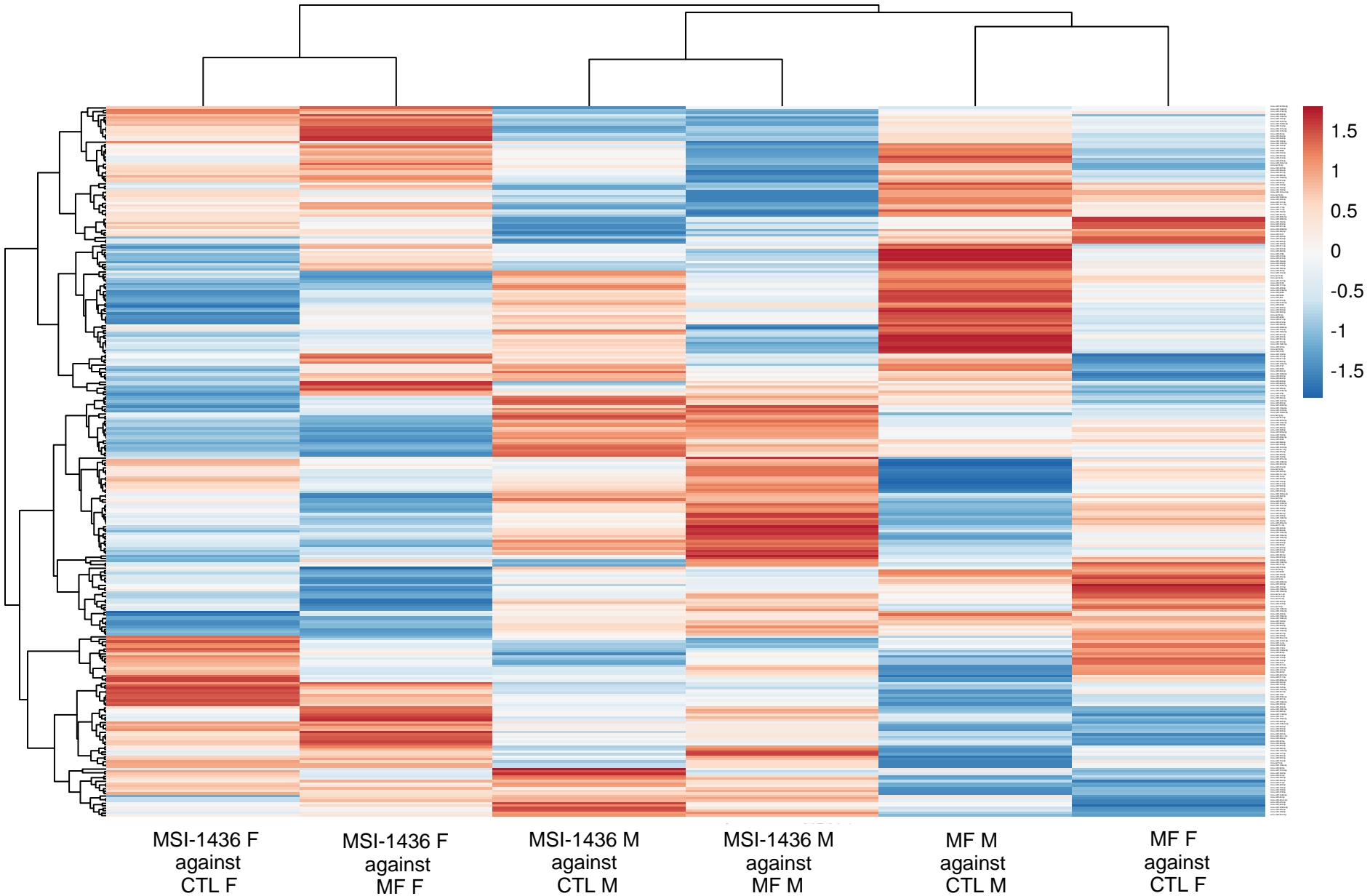

Supplement: Supplementary file 2 — Appendix S2. [file ACEL-23-e14227-s001.zip › acel14227-sup-0004-AppendixS4.pdf]
