# Supplementary material for: Early life interventions metformin and trodusquemine metabolically reprogram the developing mouse liver through transcriptomic alterations
Source: Aging Cell. 2024 May 27;23(9):e14227. doi: 10.1111/acel.14227 (PMC11488326; doi:10.1111/acel.14227)
Supplement: Supplementary file 3 — Appendix S3. [file ACEL-23-e14227-s003.docx]

| **Molecular Target** | **Effect on target cell/organ** | **Disease** |
| --- | --- | --- |
| Cellular membranes | Displacement of misfolded proteins (alpha-synuclein, beta amyloid, TDP-43) (*1-4*) | Aging related diseases, PD, AD |
| Cellular membranes | Inhibition of misfolded protein aggregation(*1-4*) | Aging related diseases, PD, AD, FTD |
| Cellular membranes | Increase in membrane strength, fluidity, lipid distribution(*3, 5, 6*) | Aging related diseases, PD, AD, FTD |
| Cellular membranes | Prevention of membrane damage by toxic protein aggregates(*1-5, 7-10*) | Aging related diseases, PD, AD, FTD |
| Unknown | RBM3 induction (synaptogenesis) (unpublished) | Cognitive impairment/AD |
| PTP1B | Reversal of inflammation, neuronal loss, inflammation, tau phosphorylation and extension of healthy lifespan in Tau AD model (unpublished) | Aging related diseases, PD, AD, FTD |
| PTP1B | Reversal of glial inflammation and neuronal loss in a beta amyloid AD model (HAPP-J20)(*11*) | Aging related diseases, PD, AD, FTD |
| PTP1B | Improved synaptic plasticity in hippocampus(*12*) | AD, PD, age-related cognitive impairment |
| PTP1B | Sensitization of Insulin receptor(*13-18*) | AD, PD, age-related cognitive impairment, Type 2 Diabetes, obesity |
| PTP1B | Sensitization of Leptin Receptor(*14, 19*) | AD, PD, age-related cognitive impairment, Type 2 Diabetes, obesity |
| PTP1B | Stimulation of amygdalar endocannabinoid release(*20, 21*) | Anxiety, AD, PD |
| PTP1B | Mobilization of hepatic lipids(*18, 19*) | Fatty liver disease, NASH, NAFLD |
| PTP1B | Reduction in ER stress(*18, 22-25*) | Aging related diseases |
| PTP1B | Improvement in mitochondrial function(*23-26*) | Aging related diseases |
| PTP1B | Inhibits PTP1B oncogenic activity(*27, 28*) | Breast Cancer |
| PTP1B | Stimulates T cell anti-tumor immunity(*28*) | Aging related diseases, Cancer |
| PTP1B | Restores flow induced arteriole dilatation (*22*) | Cerebrovascular disease |
| PTP1B | Reverses pre-existing atheroma(*29*) | Cardiovascular disease and stroke |
| PTP1B | Prevents aortic valve calcification(*26*) | Valvular heart disease |
| PTP1B | Mobilizes cardiac stem cells post myocardial infarction and inhibits fibrotic repair(*30*) | Myocardial infarction |
| PTP1B | Mobilizes skeletal muscle stem cells following traumatic injury(*30*) | Sarcopenia, traumatic injury |

Supplemental Table 8: Summary of findings in existing literature on MSI-1436. (PD: Parkinson’s disease; AD: Alzheimer’s disease; FTD: frontotemporal dementia; NASH: non-alcoholic steatohepatitis; NAFLD: non-alcoholic fatty liver disease)

1. M. Perni *et al.*, Multistep Inhibition of alpha-Synuclein Aggregation and Toxicity in Vitro and in Vivo by Trodusquemine. *ACS Chem Biol* **13**, 2308-2319 (2018).

2. R. Limbocker *et al.*, Squalamine and Its Derivatives Modulate the Aggregation of Amyloid-beta and alpha-Synuclein and Suppress the Toxicity of Their Oligomers. *Front Neurosci* **15**, 680026 (2021).

3. R. Limbocker *et al.*, Squalamine and trodusquemine: two natural products for neurodegenerative diseases, from physical chemistry to the clinic. *Nat Prod Rep* **39**, 742-753 (2022).

4. R. Limbocker *et al.*, Trodusquemine enhances Abeta42 aggregation but suppresses its toxicity by displacing oligomers from cell membranes. *Nat Commun* **10**, 225 (2019).

5. S. Errico *et al.*, Making biological membrane resistant to the toxicity of misfolded protein oligomers: a lesson from trodusquemine. *Nanoscale*, (2020).

6. B. Barletti *et al.*, Reorganization of the outer layer of a model of the plasma membrane induced by a neuroprotective aminosterol. *Colloids Surf B Biointerfaces* **222**, 113115 (2023).

7. M. Perni *et al.*, A natural product inhibits the initiation of alpha-synuclein aggregation and suppresses its toxicity. *Proc Natl Acad Sci U S A* **114**, E1009-E1017 (2017).

8. R. Limbocker *et al.*, Trodusquemine displaces protein misfolded oligomers from cell membranes and abrogates their cytotoxicity through a generic mechanism. *Commun Biol* **3**, 435 (2020).

9. S. Errico *et al.*, Quantitative Attribution of the Protective Effects of Aminosterols against Protein Aggregates to Their Chemical Structures and Ability to Modulate Biological Membranes. *J Med Chem*, (2023).

10. S. Errico *et al.*, Quantitative Measurement of the Affinity of Toxic and Nontoxic Misfolded Protein Oligomers for Lipid Bilayers and of its Modulation by Lipid Composition and Trodusquemine. *ACS Chem Neurosci* **12**, 3189-3202 (2021).

11. K. M. Ricke *et al.*, Neuronal Protein Tyrosine Phosphatase 1B Hastens Amyloid beta-Associated Alzheimer's Disease in Mice. *J Neurosci* **40**, 1581-1593 (2020).

12. L. Zhang *et al.*, Tyrosine phosphatase PTP1B impairs presynaptic NMDA receptor-mediated plasticity in a mouse model of Alzheimer's disease. *Neurobiol Dis* **156**, 105402 (2021).

13. R. S. Ahima *et al.*, Appetite suppression and weight reduction by a centrally active aminosterol. *Diabetes* **51**, 2099-2104 (2002).

14. K. A. Lantz *et al.*, Inhibition of PTP1B by trodusquemine (MSI-1436) causes fat-specific weight loss in diet-induced obese mice. *Obesity (Silver Spring)* **18**, 1516-1523 (2010).

15. R. Maccari, R. Ottana, Can Allostery Be a Key Strategy for Targeting PTP1B in Drug Discovery? A Lesson from Trodusquemine. *Int J Mol Sci* **24**, (2023).

16. M. Zasloff *et al.*, A spermine-coupled cholesterol metabolite from the shark with potent appetite suppressant and antidiabetic properties. *Int J Obes Relat Metab Disord* **25**, 689-697 (2001).

17. N. R. Pandey *et al.*, LMO4 is required to maintain hypothalamic insulin signaling. *Biochem Biophys Res Commun* **450**, 666-672 (2014).

18. L. Bourebaba, A. Serwotka-Suszczak, N. Bourebaba, M. Zyzak, K. Marycz, The PTP1B Inhibitor Trodusquemine (MSI-1436) Improves Glucose Uptake in Equine Metabolic Syndrome Affected Liver through Anti-Inflammatory and Antifibrotic Activity. *Int J Inflam* **2023**, 3803056 (2023).

19. N. Takahashi, Y. Qi, H. R. Patel, R. S. Ahima, A novel aminosterol reverses diabetes and fatty liver disease in obese mice. *J Hepatol* **41**, 391-398 (2004).

20. Z. Qin *et al.*, Chronic stress induces anxiety via an amygdalar intracellular cascade that impairs endocannabinoid signaling. *Neuron* **85**, 1319-1331 (2015).

21. N. Krishnan, N. K. Tonks, Anxious moments for the protein tyrosine phosphatase PTP1B. *Trends Neurosci* **38**, 462-465 (2015).

22. P. A. Thiebaut *et al.*, Protein tyrosine phosphatase 1B regulates endothelial endoplasmic reticulum stress; role in endothelial dysfunction. *Vascul Pharmacol* **109**, 36-44 (2018).

23. L. Bourebaba *et al.*, The PTP1B inhibitor MSI-1436 ameliorates liver insulin sensitivity by modulating autophagy, ER stress and systemic inflammation in Equine metabolic syndrome affected horses. *Front Endocrinol (Lausanne)* **14**, 1149610 (2023).

24. L. Bourebaba, S. S. B. Komakula, C. Weiss, N. Adrar, K. Marycz, The PTP1B selective inhibitor MSI-1436 mitigates Tunicamycin-induced ER stress in human hepatocarcinoma cell line through XBP1 splicing modulation. *PLoS One* **18**, e0278566 (2023).

25. L. Bourebaba *et al.*, MSI-1436 improves EMS adipose derived progenitor stem cells in the course of adipogenic differentiation through modulation of ER stress, apoptosis, and oxidative stress. *Stem Cell Res Ther* **12**, 97 (2021).

26. F. Liu *et al.*, PTP1B Inhibition Improves Mitochondrial Dynamics to Alleviate Calcific Aortic Valve Disease Via Regulating OPA1 Homeostasis. *JACC Basic Transl Sci* **7**, 697-712 (2022).

27. N. Krishnan *et al.*, Targeting the disordered C terminus of PTP1B with an allosteric inhibitor. *Nat Chem Biol* **10**, 558-566 (2014).

28. F. Wiede *et al.*, PTP1B Is an Intracellular Checkpoint that Limits T-cell and CAR T-cell Antitumor Immunity. *Cancer Discov* **12**, 752-773 (2022).

29. D. Thompson *et al.*, Pharmacological inhibition of protein tyrosine phosphatase 1B protects against atherosclerotic plaque formation in the LDLR(-/-) mouse model of atherosclerosis. *Clin Sci (Lond)* **131**, 2489-2501 (2017).

30. A. M. Smith *et al.*, The protein tyrosine phosphatase 1B inhibitor MSI-1436 stimulates regeneration of heart and multiple other tissues. *NPJ Regen Med* **2**, 4 (2017).
